# Vascular Perspectives of the Midfacial Superficial Musculoaponeurotic System

**DOI:** 10.3390/diagnostics14202294

**Published:** 2024-10-16

**Authors:** Delia Hînganu, Marius Valeriu Hînganu, Camelia Tamaș, Victor Vlad Costan, Liliana Hristian, Dragoș Negru, Anca Elena Calistru, Ramona Paula Cucu, Ludmila Lozneanu

**Affiliations:** 1 Department of Morpho-Functional Sciences I, Faculty of Medicine, “Grigore T. Popa” University of Medicine and Pharmacy, 700115 Iasi, Romania; hinganu.delia@umfiasi.ro (D.H.); ludmila.lozneanu@umfiasi.ro (L.L.); 2 Department of Plastic and Reconstructive Surgery, “Grigore T. Popa” University of Medicine and Pharmacy, 700115 Iasi, Romania; camelia.tamas@umfiasi.ro; 3 Department of Oral and Maxillo-Facial Surgery, “Grigore T. Popa” University of Medicine and Pharmacy, 700115 Iasi, Romania; victor.costan@umfiasi.ro (V.V.C.); ramona_cucu@umfiasi.ro (R.P.C.); 4 Department of Engineering and Design of Textile Products, “Gheorghe Asachi” Technical University of Iasi, 700050 Iasi, Romania; liliana.hristian@academic.tuiasi.ro; 5 Department of Radiology, Faculty of Medicine, “Grigore T. Popa” University of Medicine and Pharmacy, 700115 Iasi, Romania; dragos.negru@umfiasi.ro; 6 Department of Pedotechnics, “Ion Ionescu de la Brad” University of Life Sciences, 700490 Iasi, Romania; aecalistru@uaiasi.ro

**Keywords:** SMAS, MRA, vascular microperfusion of face, endothelium, collagen

## Abstract

**Objectives**: Presently, data on the vascularization of the superficial musculoaponeurotic system of the face (SMAS) are lacking. Thus, the present study aimed to provide new conclusive data about the topography, density, and relationship of the SMAS blood vessels with other components, namely, the fibrous connective tissue and muscles. **Methods**: The study included a control lot of 42 cases from the archive of the radiology department. In this group, nuclear magnetic resonance angiography (MRA) was performed in order to identify the main sources of vascular supply. In the second group, tissue samples were collected from the midfacial region of 45 patients from the Oro-Maxillo-Facial and Plastic and Reconstructive Surgery clinics of ‘St. Spiridon’ County Clinical Emergency Hospital, Iasi. These patients received surgery for excision of tumoral formations that did not involve SMAS components. These samples underwent micro-CT analysis, hematoxylin and eosin (HE) staining, as well as immunohistochemical (IHC) staining for collagen type III, muscle tissue, and the vascular endothelium. **Results**: We discovered the particular way in which the SMAS components interrelate with vascularization and the regional differences between them. We have discovered a new vascular network specific to the SMAS, highlighted by both the micro-CT technique and microscopy on slides with special IHC staining. Significant differences were observed in the topographic arrangement, density, and relationships of the microscopic vasculature across midfacial regions. IHC staining provided morphological and functional information about the structure and vascularization of SMAS. **Conclusions**: The MRA technique could not detect the structural blood vessels of the SMAS and other methods for their in vivo visualization must be sought. The blood vessels of the SMAS mainly follow the topography of the muscle fibers. From the SMAS layer where they are found, the distribution branches reach the stroma of the region and the hypoderm. Our data can contribute to the development of surgical techniques tailored to each individual patient, as well as the enhancement of methods for stimulating cutaneous angiogenesis, improving scarring in this region, and advancing biotissue engineering techniques.

## 1. Introduction

Detailed knowledge of the architecture of the topographical layers of the face is essential for the success of facial aesthetic surgical procedures. Understanding the complex surgical anatomy of the face leads to safe and effective outcomes. The concept of a single superficial subcutaneous layer is highly important, both anatomically and in reconstructive surgical practice [1,2]. This layer is known as the superficial musculoaponeurotic system of the face (SMAS).

The topographic study of the SMAS highlights regional specific features related to the muscles, blood vessels, and nerves, as well as to the presence of fixation manners (retaining structures) of the superficial fascia. The essential part of the SMAS is the superficial fascia, which continues in all regions of the face. The blood vessels and ramifications of the terminal branches of the facial and trigeminal nerves cross several regions of the face in close connection with the superficial fascia. The superficial fascia protects the ramifications of blood vessels and nerves by ensheathing them. Meanwhile, the SMAS layer is crossed by the cutaneous and proper vasculonervous branches [3], which can guarantee the success of skin grafting.

The SMAS is ‘an amplifier of facial muscle contraction’. It acts as a distributor of muscle contraction force vectors to the skin; each muscle contraction follows a preferential direction in this musculoaponeurotic network. An infinite number of actions are possible because the SMAS distributes muscle contractions along the parallel network to the skin plane and it distributes the resulting effects in a direction perpendicular to the skin through fibrous expansions from the SMAS to the dermis.

Additionally, the soft tissue planes are supported in the normal anatomical position by a series of retaining manners that come from deep fascia, fixing the facial structures to the overlying dermis [4]. The nasal SMAS forms part of the SMAS of the face, as a continuous fibromuscular layer interconnecting the mimic muscles and surrounding regional fascia. Its function is to change the transnasal pressure of the nasal valve during respiration [5]. Furthermore, its corresponding subcutaneous fat is concentrated on the glabella, nasal wing, and tip of the nose. Moreover, the distribution of sub-SMAS fat is reported to be similar to that of supra-SMAS fat [6,7].

Ghassemi et al. identified two histologically distinct types of the SMAS located laterally and medially to the nasolabial fold. The first type, found lateral to the nasolabial fold, features relatively small fibrous septa that encase fat cell lobules, while the second type, situated medial to the nasolabial fold, consists of a dense collagen–muscle fiber meshwork [8,9]. The primary blood supply to the nasal region and nasolabial fold comes from the facial and ophthalmic arteries. Variations in the facial artery’s location, direction, and branching are common; it may run directly toward the oral commissure and into the upper lip medially to the nasolabial fold, which is the most frequent distribution pattern, or it may travel into the cheek, lateral to the nasolabial fold, branching into the lateral nasal and angular arteries. Conversely, the trajectory and relationships of the ophthalmic artery have not been reported to vary in the literature. The vascularization of the nasal SMAS arises from a polygonal, multidirectional anastomotic system formed by three transfacial arcades (alar, valve, and radix), connected by other longitudinal arteries (angular, dorsal, and intermediate). The nasal SMAS comprises a superficial fascia and a subcutaneous adipose layer (supra-SMAS). On the ala nasi, mimic muscles attach to the dermis transfascially, forming a clear delineation as the SMAS line, whereas the superficial fascia in the nasal dorsum lacks muscular fascicles. Despite the wealth of information on the morphology and topography of this morphofunctional feature, existing data on the microvascularization of the nasal SMAS remain limited [10,11,12,13].

The complex processes of wrinkle healing as well as the physiological mechanisms that fight against the phenomenon of ‘facial aging’ are influenced by the quality of regional vascularization. Moreover, significant recent advances in the field of tissue bioengineering to improve the vascularization of neotissue have been made for repairing or regenerating accidental or postoperative wounds [14]. All of these studies must be based on the morphofunctional characteristics of the considered regions and the particular primordial aspects of their vascularization.

Recent studies have shown that the fields of facial reconstruction as well as tissue engineering have evolved significantly, developing various strategies to accelerate and improve skin regeneration. However, there are still many concerns related to the existing limitations regarding vasculature, which is known as the most important challenge [7]. An insufficient or functionally compromised vascular network ultimately results in ischemia [15].

This study aimed to provide important topographic and morphologic information about the nasal SMAS vascular apparatus from an integrated radiological, histological, and immunohistochemistry (IHC) perspective. We sought to investigate the morphology of the nose from a relatively new perspective of a continuous superficial layer that is closely related to both the integument of the region and the subjacent muscle layer. Our results will have direct applicability in rhinoplasty techniques, nasal reconstructions, and new conservative dissection protocols.

## 2. Materials and Methods

Our study was conducted in three different research centers in Iasi between January 2020 and February 2024. The radiologic magnetic resonance angiography (MRA) study was performed at Arcadia Hospital using a 3.0 T Philips Ingenia magnetic resonance imaging (MRI) scanner. The micro-CT study was conducted at the ICAM Iasi (Research Institute for Agriculture and the Environment), within the ‘Ion Ionescu de la Brad’ University of Life Sciences in Iaşi. Histological and IHC studies were performed in the Pathological Anatomy laboratory of the ‘St. Spiridon’ Emergency Clinical Hospital in Iasi.

Tissue blocks were obtained from nasal and regions during surgical procedures for different pathologies that did not involve the quality of SMAS tissues. The exclusion criteria were extensive facial malignant tumors of the skin and subcutaneous tissues, those with oncologic resections, all types of dermatologic conditions, history of radiation therapy, children, pregnant women, and people with systemic vascular diseases. The ages of the men and women ranged from 18 to 72 years and from 18 to 73 years, respectively.

The present study followed the principles outlined in the Declaration of Helsinki. Written informed consent was obtained from each participant who underwent investigation. The ethics committee approval of ‘Grigore T. Popa’ University of Medicine and Pharmacy Iasi number 195, dated 3 June, 2022, is attached to this manuscript.

For this study, we included two groups of patients: the first group underwent MRA, while the second group was designated for histological, immunohistochemical, and micro-CT analyses. All tissue samples from the second group were divided into two parts: one portion, preserved in formalin solution, was used for the micro-CT study, while the other, preserved in 10% paraffin, was used for histological and immunohistochemical analyses.

### 2.1. Radiologic Study

#### 2.1.1. MRA Study

For the first study group, we selected 42 cases from the archive of the radiology department. These patients were examined by performing nuclear magnetic resonance angiography (MRA) using a Philips MR Ingenia Elition 3.0 T device. MRA—sometimes called magnetic resonance angiography—is a magnetic resonance procedure that focuses on blood vessels. Cases were selected from those recommended for neurological evaluation or those with preoperative medical conditions that did not interfere with midfacial SMAS vascularization and integrity.

MRA of the peripheral vasculature can be performed quickly and accurately, with a couple of distinct advantages over computed tomography angiography (CTA), which are as follows: it does not use a nephrotoxic contrast agent and patients are not exposed to radiation. The quality of the images obtained using MRA is so good that it has virtually surpassed angiography for evaluating stable patients with PAD and it can assess vessel occlusion, stenosis, and the arterial wall [16]. We used time-of-flight angiography (TOF) to visualize blood flow within vessels [17]. The identification of blood vessels by this method can sometimes be difficult because they are relatively hard to differentiate from the surrounding tissue, with a predominance in T1 weighting. Maximum intensity projection (MIP) is a simple three-dimensional visualization tool that can be used to display CTA datasets. MIP images are not threshold dependent, preserve attenuation information, and represent a popularly used algorithm for the display of MRA images. Concurrently, the vessel-missing probability, vessel receiver operating characteristics, and mean number of false vessels are also considered. Based on the assumption that the intensities of vessel, tissue, and noise along a projection path are independent and Gaussian, these measures are derived and obtained in closed forms.

Increasing TR is often undesirable because the signal from the background tissue also increases, with loss of vessel conspicuity. Moreover, increasing TR also lengthens the scan time. Thus, we tried to keep the TE as short as possible to keep the slices as thin as possible whenever moderating TR and flap angle choices. The presented images were selected from TR = 11 (repetition time) and TE = 4.6 (time to echo).

In our study, we considered s3DI_MC_HR (three-dimensional high-resolution time-of-flight—TOF—used in MR angiography acquisition to visualize flow within arterial vessels), SAG/COR (sagittal and coronal planes), and TRA (transverse plane) MIP (maximum intensity projection) for data acquisition.

Using this radiological technique, we identified the main blood vessel sources in the nasal and oral regions. MRA allows for ideal skeletal topographic reporting of tissue sampling points. At the same time, these techniques allow for the objectification of the origin of blood vessels found on IHC slides and µCT sections.

#### 2.1.2. Micro-CT Study

Tissue samples were collected from the same patients enrolled in the histological and IHC studies. This second study group comprised 45 patients (27 specimens from nasal region and 18 from oral region) from the Oro-Maxillo-Facial and Plastic and Reconstructive Surgery clinics of ‘St. Spiridon’ County Clinical Emergency Hospital, Iasi, from whom tissue fragments were collected from the ala nasi and nasolabial fold. Tissue samples from the second study group were collected during surgical procedures that preserved the integrity of the SMAS. The incision was made perpendicular to the dermis, extending to the level of the alar cartilage without penetrating it. These patients received surgery for excision of tumoral formations that did not involve SMAS components.

Within this group, samples were collected from the level of the nasal pyramid, the wing of the nose, and from the level of the medial part of the nasolabial fold.

This study used a Bruker SKYSCAN 1273 High Capacity 3D X-ray microscope equipped with a large-format 6 MP detector based on the latest flat-panel technology, achieving very high contrast in the accumulated images owing to its large dynamic range. The fast frame rate along with an optimized scintillator enables excellent image quality in a stunningly short cycle of <15 s, which is ideal for time-resolved 3D X-ray microscopy.

Micro-CT differentiation of tissue density is non-destructive. The object size of 250 mm in diameter and 250 mm in height confers the capability to scan huge samples in a benchtop instrument. Bruker SKYSCAN 1273 icluded 3D.SUITE software needed to analyze data, as follows: NRecon version 2.1.0.1, which transforms the 2D projection images into 3D volumes; analysis software, DATAVIEWER, version 1.5.6.5, which performs slice-by-slice inspection of 3D volumes and 2D/3D image registration, CTvox, version 3.3, which performs realistic visualization by volume rendering, and CTAN version 1.20.8.0, which performs 2D/3D image analysis and processing.

We adjusted the study protocol following a series of tests on tissue samples fixed in 10% paraffin and tissue samples fixed in formalin solutions. The paraffin-fixed samples did not yield the expected results, likely due to their insufficient thickness (<10 mm) and the low radiopacity of both the tissue and the fixation medium.

The tissue samples fixed in formaline were transferred into a 7.5% Lugol solution. Lugol’s iodine, also known as an aqueous and strong iodine solution, is a solution of potassium iodide with iodine in water. These parts were analyzed with the micro-CT device at 4, 7, and 14 days after immersion in the Lugol solution. We also analyzed the tissue samples at 7 and 20 µm [18].

Scanning was performed using the samples removed from the Lugol solution and mechanically fixed in a support consisting of a plastic container filled with polypropylene fragments. Owing to the reduced scanning time of approximately 20 min, drying of the samples was avoided.

### 2.2. Histological and Immunohistochemical Analyses

The specimens were preserved in paraffin and then examined by special immunohistochemistry (IHC) and HE techniques. These techniques included the parallel use of an IHC marker for muscle tissue, MyoH2, for type III collagen and I-CAM2 for angiogenesis phenomena and endothelial cells. These markers were scored between 0 and 3, according to a histological scoring system, following the amount of collagen fibers, epithelial cells, and muscle fibers, as well as the intensity of staining. The subjective qualitative assessment was graded as follows: (0) for negative results; (1) + small amounts/low (weak) intensity; (2) ++ moderate amounts/moderate intensity; and (3) +++ large amounts/strong intensity) [19]. Each type of area had a characteristic staining pattern for collagen type III, ICAM-2, and MyoH2.

Each section of tissue had a positive control (normal connective tissue elements, endothelial cells, and muscle fibers). All 45 human sections were stained with collagen III, ICAM-2, and MyoH2 antibodies.

Formaldehyde-fixed human tissue was embedded in paraffin wax, and the dewaxed sections were incubated in phosphate-buffered saline and cut in 4 μm sections for IHC staining. Heat-induced epitope retrieval with citrate buffer (pH 6.0) was performed before peroxide blocking for 10 min and staining with collagen type III (clone FH-7A, Abcam, Cambridge, UK), 1/500 dilution; ICAM-2 (clone EPR 19114-113), 1/4000 dilution, Abcam, Cambridge, UK); and MyH2 (clone A4.74, Abcam, Cambridge, UK), 1/1000 dilution. The sections were developed using mouse- and rabbit-specific HRP/DAB detection IHC kits with HRP-conjugated secondary antibodies (biotinylated goat anti-polyvalent) for 10 min, streptavidin peroxidase, and counterstained with haematoxylin to visualize the morphology of the collagen fibers, endothelial cells, or muscle cells. The images of the histological slides were taken at different magnifications with a camera attached to a light microscope (LEICA DM3000 LED, manufactered by Leica Microsystems in Wetzlar, Germany) using a LEICA MC 190 HD lens, manufactered by Leica Microsystems in Wetzlar, Germany.

### 2.3. Statistics Analyses

To decide whether the independent variables included in the study follow a normal distribution, researchers [20,21] recommend transforming the two coefficients into z-scores.

If one of the z scores thus obtained is bigger than 1 (or 1.5 for small volume samples), then the distribution differs significantly from a standard, regular one.

The verification of the normality of the distribution of the results obtained for each independent variable (collagen III, ICAM-2, MyoH2, LTD, LVD, LBV/VHD) according to the dependent variable, anatomical region, was carried out by graphic and statistical methods in the SPSS Statistics Version 20 program (IBM SPSS Statistics software 29) using the Kolmogorov–Smirnov test and the Shapiro–Wilk test.

The study included:

- Numerical independent variables:
  o LTD = Largest Transverse Diameter
  o LVD = Largest Vessel Diameter
  o LBV/VHD
  o VHD = Vascular Hillum Diameter—only applicable to labial measurements
  o LBV = Length of the Blood Bessel = applicable only to the measurements from the nasal region
- Categorical independent variables:
  o Collagen III ((1) + small amounts/low intensity (weak); (2) ++ moderate amounts/moderate intensity; (3) +++ large amounts/strong intensity)
  o ICAM-2((1) + small amounts/low intensity (weak); (2) ++ moderate amounts/moderate intensity; (3) +++ large amounts/strong intensity)
  o MyoH2((1) + small amounts/low intensity (weak); (2) ++ moderate amounts/moderate intensity; (3) +++ large amounts/strong intensity)
  o Categorical dependent variable, anatomical region (nasal and oral)

We also used the Shapiro–Wilk (S–W) test from a normally distributed population and the Kolmogorov–Smirnov test to estimate the normality of the distribution where the mean and standard deviation could be calculated. These tests are used to test the hypothesis that a data sample follows a certain distribution law, as well as to compare the distribution laws of the populations from which two samples come [22,23,24,25,26,27,28,29,30,31].

## 3. Results

All of our study data are presented in the mentioned order of the work research methodology.

### 3.1. MRA Study Results

**Figure 1:**
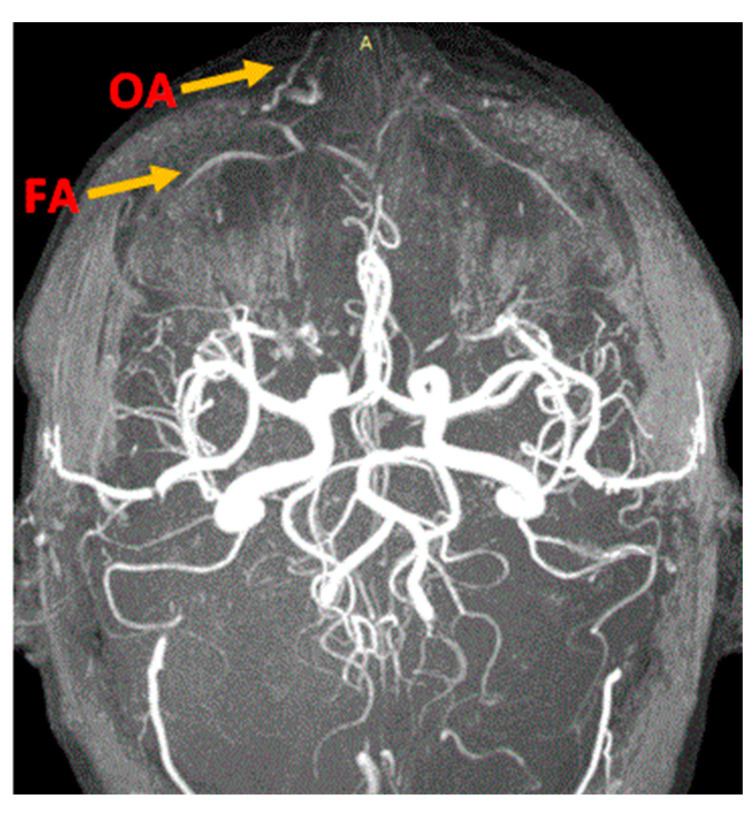
Branches of facial (FA) and ophthalmic arteries (OA) for the nasal region. MRA VPRV 18 yo healthy patient. 9 min and 13 s time of exposure. A: Artery.

**Figure 2:**
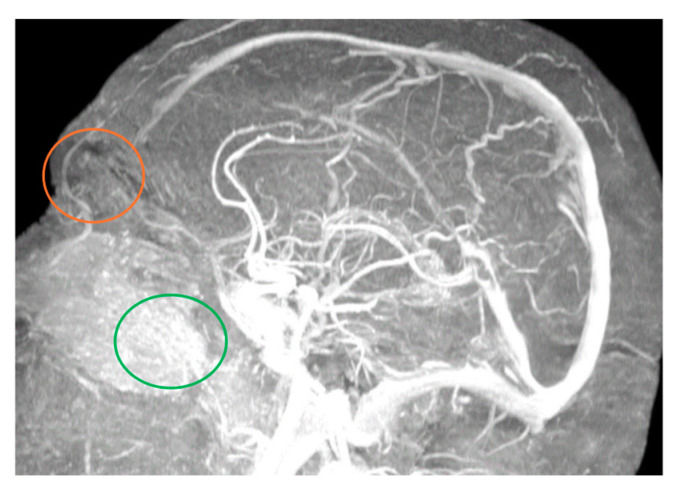
Branches from supraorbital and ophthalmic arteries (orange circle), as well as facial artery terminal nasal branches (green circle). DT 51 yo patient. 12 min and 24 s time of exposure.

MRA easily illuminated the main blood sources of the nasal region, even if the examination protocol was not dedicated to the exploration of this region. We could identify the branches of the type 1 facial and ophthalmic arteries of the nasal region (Figure 1 and Figure 2) and at least one branch coming from the supraorbital artery (Figure 2).

**Figure 3:**
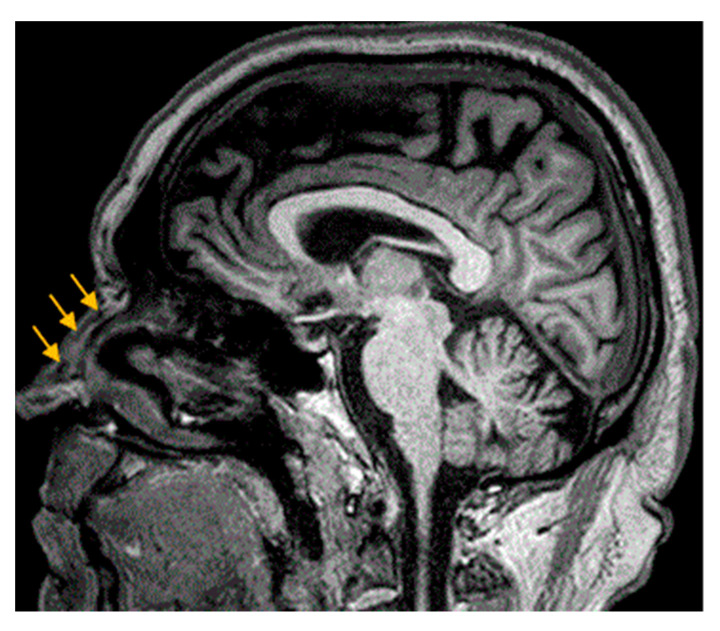
Nasal SMAS (orange arrows). TM 68 yo patient. 7 min and 30 s time of exposure.

We identified the individual nasal SMAS layer and its continuity with the glabellar (frontal) fascia (Figure 3). The SMAS is a well-marked morphological entity at the wing level of the nose and nasal pyramid, but it is gradually lost and descends toward its tip. The adipose tissue is present in the sub-SMAS layer and has a compact appearance. However, MRA did not provide concrete data on SMAS microvascularization.

**Figure 4:**
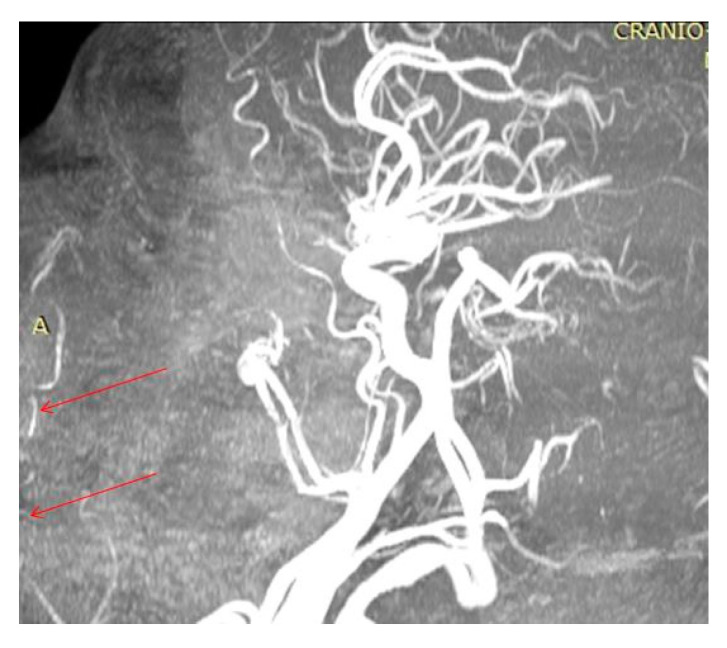
Identification of the superior (superior red arrow) and inferior (inferior red arrow) labial branches of the facial artery (A).

In the oral region, at the level of the lips, the main vascularization sources were objectified—branches from the facial artery (Figure 4). These blood vessels cross the labial SMAS layer and distribute to the skin and mucosa at this level.

### 3.2. Results of the Micro-CT Study

There were no significant differences in Lugol immersion between 4 and 7 days, which is why we split the results of the micro-CT study into the following four groups: 7 and 14 days of immersion into the Lugol iodide solution, each of them scanned at 7 and 20 µm.

#### 3.2.1. After 7 Days of Immersion

##### Scan at 7 µM 

**Figure 5:**
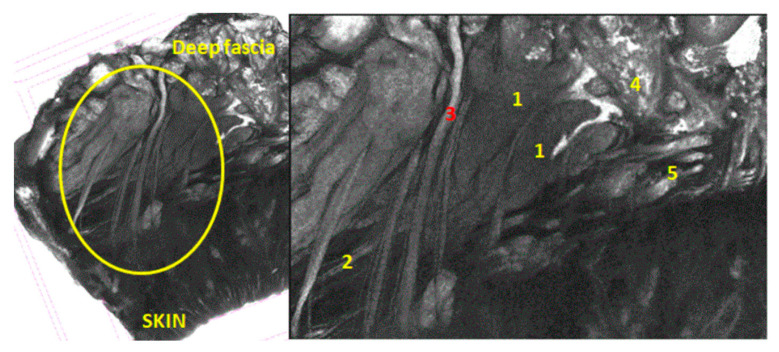
Specimen immersed in Lugol for 7 days from medial side of nasolabial groove; 7 µm scan; yellow circle = SMAS area; 1 = mimic muscles; 2 = fibrous tissue from superficial fascia; 3 = blood vessel; 4 = fat tissue; 5 = attaching manners.

After the first 7 days of immersion in the Lugol solution, the scan results showed a distinctive vascular pathway through the muscles, medially to the nasolabial fold. The blood vessel was followed by nervous fibers (Figure 5).

##### Scan at 20 µM 

**Figure 6:**
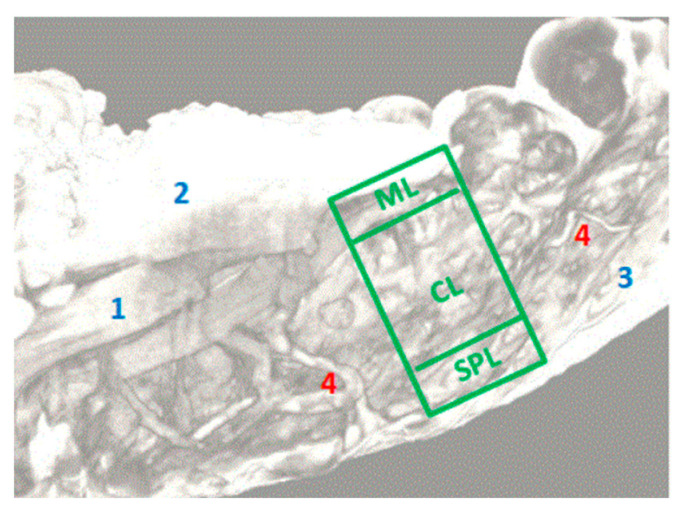
Specimen immersed in Lugol for 7 days from nasal pyramid area; 1 = mimic muscle (procerus); 2 = sub-SMAS fat tissue; 3 = superficial fascia, 4 = blood vessels; ML = muscle layer; CL = conjunctive layer; SPL = superficial fascia layer; whole green rectangular = SMAS.

On the 20 µm scan after 7 days of immersion, we were able to distinguish an entire network of blood vessels within the thickness of the SMAS in the area of the nasal pyramid (Figure 6). They cross the SMAS in a random manner—sagittally, coronary, or tortuously. There are no particular conjunctival tissue fibers that follow the blood vessels in this study. Sagittal vessels split in a “T” shape manner when they reach the skin. Therefore, they form a clearly distinguishable blood distribution system in parallel with the skin.

At the same time, we observed a mixed type of blood vessels in this area; cutaneous thick branches cross the SMAS and deep, thinner branches run within its thickness.

#### 3.2.2. After 14 Days of Immersion

##### Scane at 7 µM

**Figure 7:**
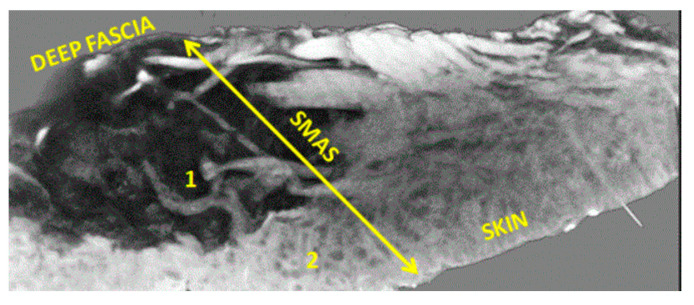
Significant proper SMAS blood vessel network (1 and 2) revealed in a specimen immersed in Lugol iodide for 14 days. Micro-CT is still able to distinguish how these vessels protrude into the thick conjunctive tissue under the skin of the tip of the nose.

**Figure 8:**
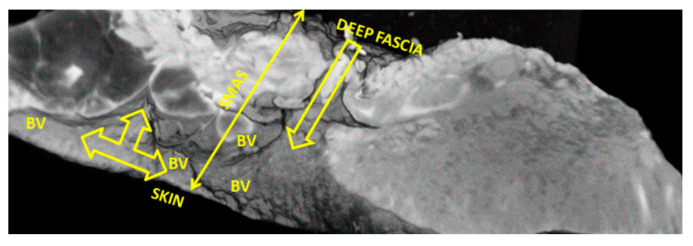
Single yellow arrow = vasculonervous pathway through SMAS; triple yellow arrow = “T” shape splitting of vasculonervous bundles, with retrograde distribution of SMAS blood vessels; BV = blood vessels.

After 14 days of immersion in the Lugol iodide solution, the image quality was slightly diminished (Figure 7 and Figure 8). The 7 µm scan still highlighted a blood vessel network right before it loses itself within the conjunctival meshwork beneath the skin (Figure 7). Deep vascular branches cross through the SMAS in this region.

##### Scan at 20 µM

The 20 µm scan showed a better image of the trans-SMAS conjunctival network of tunnels for the passage of cutaneous and proper SMAS blood vessels and nerves. They split as vasculonervous bundles in a T-shaped manner right beneath the skin. Then, these bundles follow the skin in a parallel manner (Figure 8).

**Figure 9:**
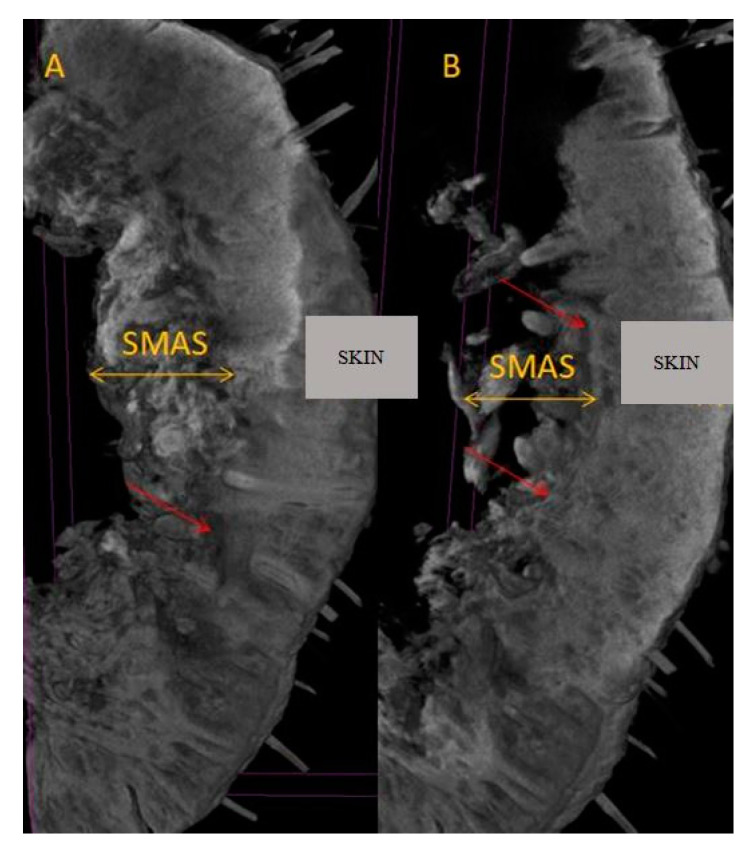
(**A**) Micro-CT at upper lip level; (**B**) micro-CT at the level of the lower lip; SMAS blood vessels are highlighted by red arrows.

After studying the results of the different immersions—7 and 14 days, respectively— we decided that in the case of the oral region, we should leave the pieces in Lugol for two weeks (Figure 9).

### 3.3. Results of the HE and IHC Studies

We have grouped the study results according to the topography of the anatomical specimens collected. During the study of the resection pieces, we applied the HE and IHC techniques.

#### 3.3.1. Nasal Pyramid

**Figure 10:**
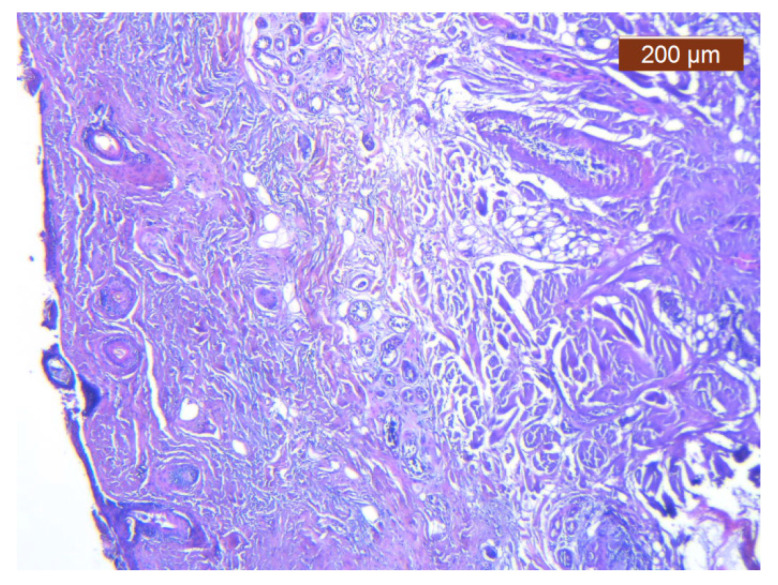
Subcutaneous collagen thick septa. Collagen fibers (deep), blood vessels, and fat cells (middle alar area) (HE ×5).

In this region, the arrangement of the SMAS layers from the superficial to the deep layers seemed very particular. Thus, in the immediate infradermal layer, we found strong collagen septa (Figure 10). The representation of blood vessels and muscle tissue at this level was almost absent.

**Figure 11:**
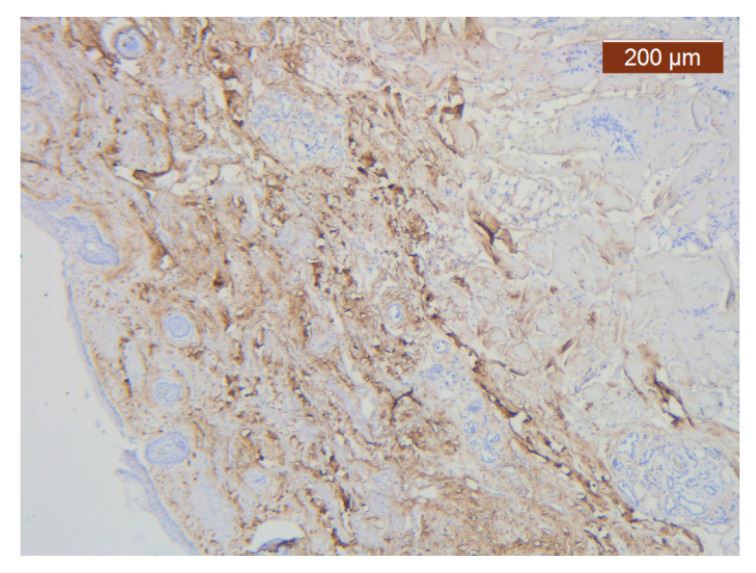
Collagen III is either absent or present in a more irregular distribution (anti-collagen III ×5). Staining intensity: strong.

**Figure 12:**
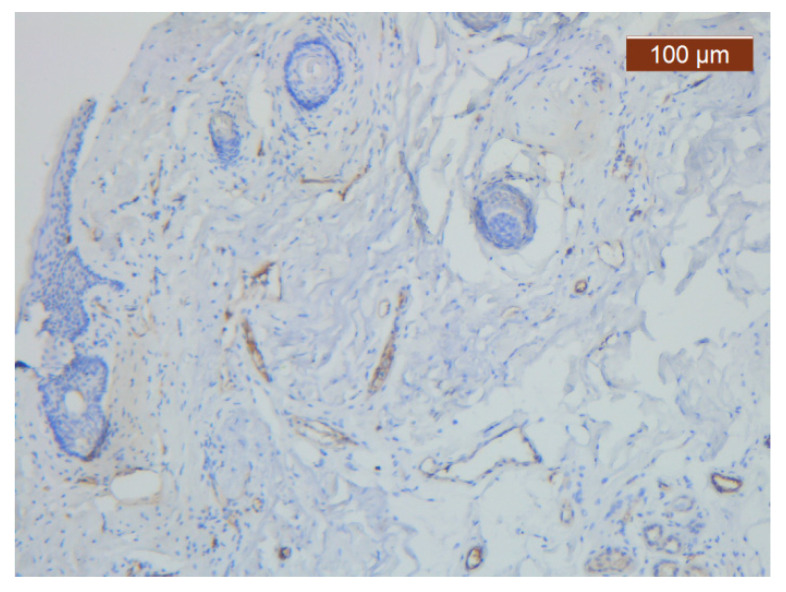
ICAM-2 staining is confined to internal vascular walls (endothelial cells) (anti-ICAM-2 ×10). Staining intensity: weak to moderate.

**Figure 13:**
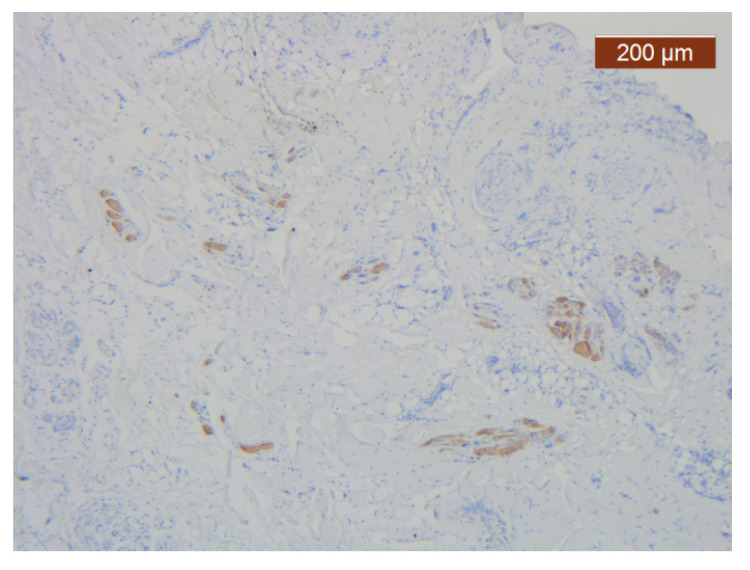
Intracytoplasmic MyH2 in muscle cells but not in the surrounding connective tissue (anti-MyH2 ×5). Staining intensity: moderate.

**Figure 14:**
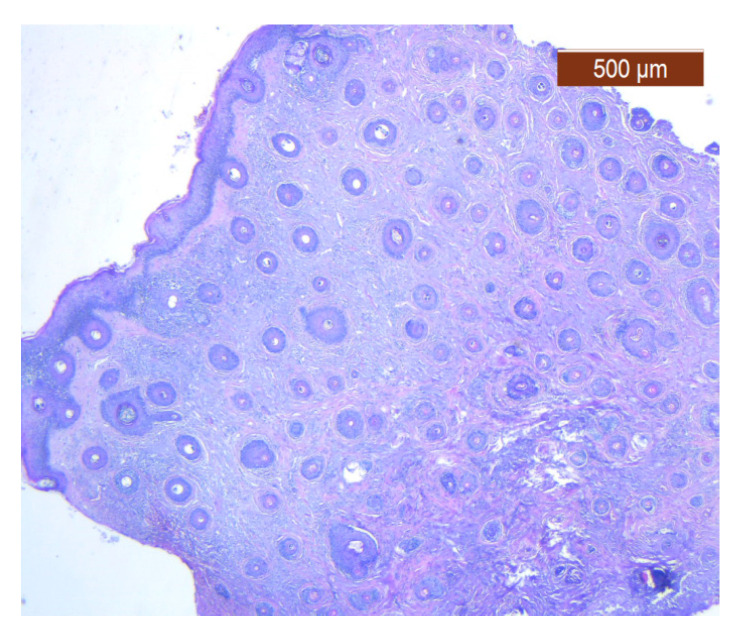
Skin with dense dermis, blood vessels, and some adipose cells (HE ×2.5).

Immediately below this layer follows an intermediate layer in which fat lobules are surrounded by collagen III fibers, with an apparently irregular arrangement, and blood vessels (Figure 11, Figure 12 and Figure 13). The muscle tissue is also present in this intermediate layer, but it was poorly represented (Figure 14).

Muscle tissue is also present in this intermediate layer but poorly represented (Figure 13).

Summarizing our results from this region, we can say that the SMAS is represented by a layer of fibrous skin adhesions, superficial and well represented, followed by an intermediate, transitional nutrient layer. This layer includes most of the blood vessels and lobulated macroadipose tissue, with fibers of the facial muscles of probable origin in the procerus muscle. In the deep layer, the three-dimensional collagen network from the transitional layer continues.

#### 3.3.2. Nasal Wing

**Figure 15:**
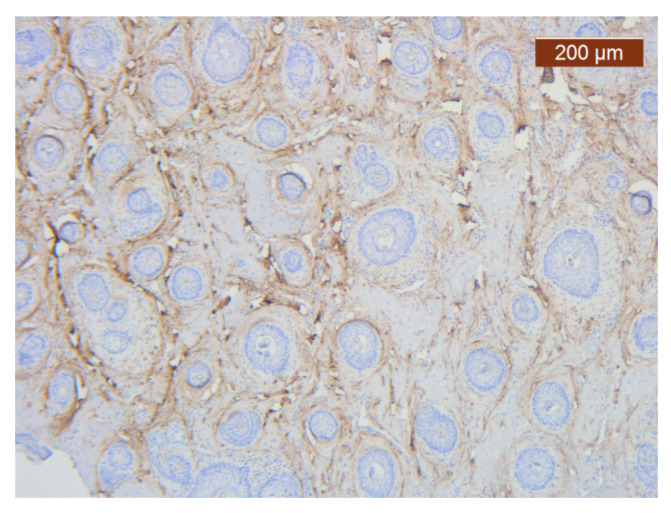
Collagen is plentiful in the stroma (anti-collagen III ×5). Staining intensity: moderate to strong.

In this region of the nose, the trilamellar topographical arrangement at the pyramid level no longer exists. Instead, we found a dense network of collagen, with fine muscle fibers seemingly randomly interspersed. The collagen fibers had a dense, peripheral appearance (Figure 14 and Figure 15).

**Figure 16:**
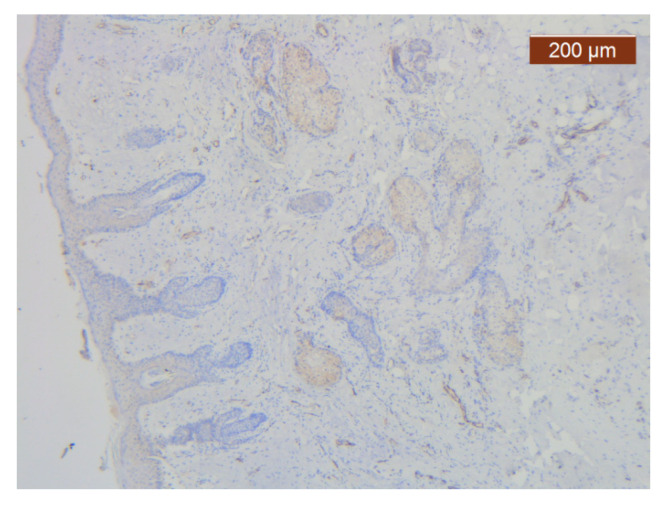
ICAM-2 moderate positivity in blood vessels (anti-ICAM-2 ×5). Staining intensity: moderate.

The vascular network of the SMAS was distributed in the same, relatively constant density throughout its thickness, up to the dermis. The transmembranar activity of the blood vessels was moderate (Figure 16).

**Figure 17:**
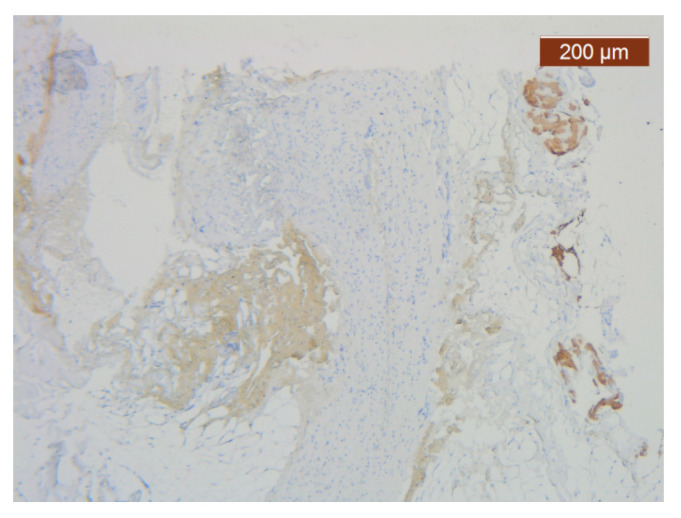
MyH2 showed irregular bundles and inegal staining distribution (MyH2 ×5). Staining intensity: moderate.

The presence of muscle fibers is moderate in density. They are organized in the form of fascicles that cross the collagen network and insert on the deep face of the dermis. Their insertion is strong, made through a layer of dense collagen, as described above (Figure 17).

#### 3.3.3. Medial to the Nasolabial Fold

**Figure 18:**
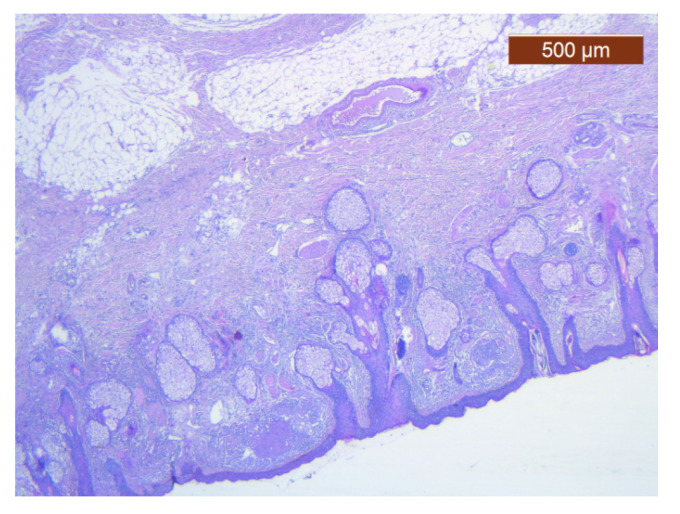
Collagen fiber meshwork and a large blood vessel with thick walls and lobules of adipose tissue (HE ×2.5).

**Figure 19:**
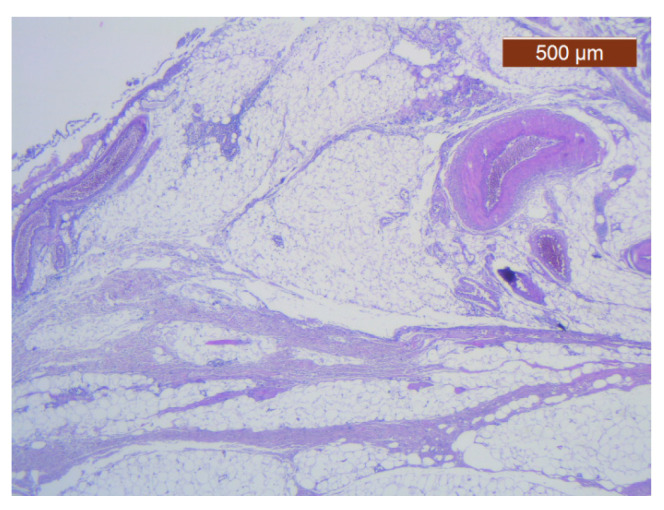
Details of collagen fiber meshwork and a large blood vessel with thick walls and lobules of adipose tissue (HE ×2.5).

**Figure 20:**
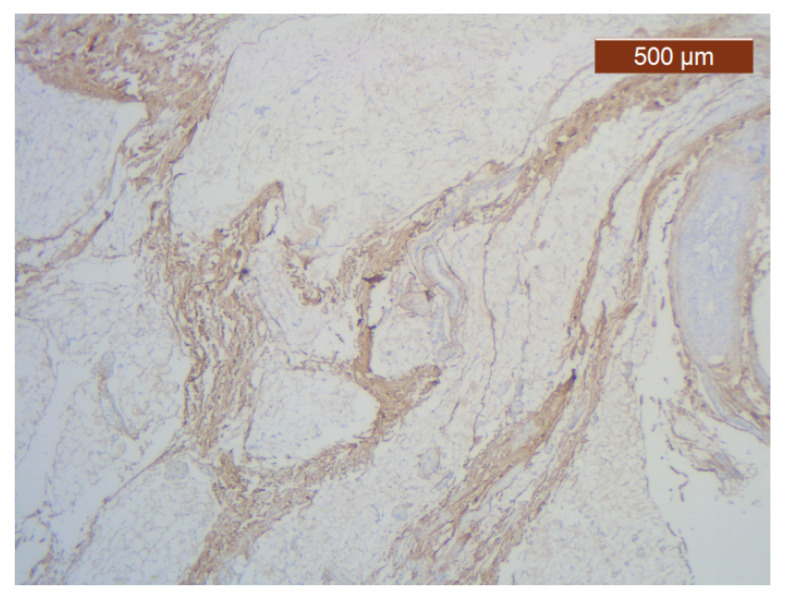
Abundant perivascuar and interstitial collagen III and around adipose cells in soft part of hypodermis (anti-collagen III ×2.5). Staining intensity: moderate to strong.

The region of the nose located medial to the nasolabial fold also presented distinct characteristics at the level of the SMAS bed. The collagen fibers had the densest appearance among the explored regions (Figure 18). This three-dimensional collagen network is distributed toward the superficial dermis and also descends into the deep dermis. At this level, it delimits and seals the large blood vessels (Figure 18, Figure 19 and Figure 20).

**Figure 21:**
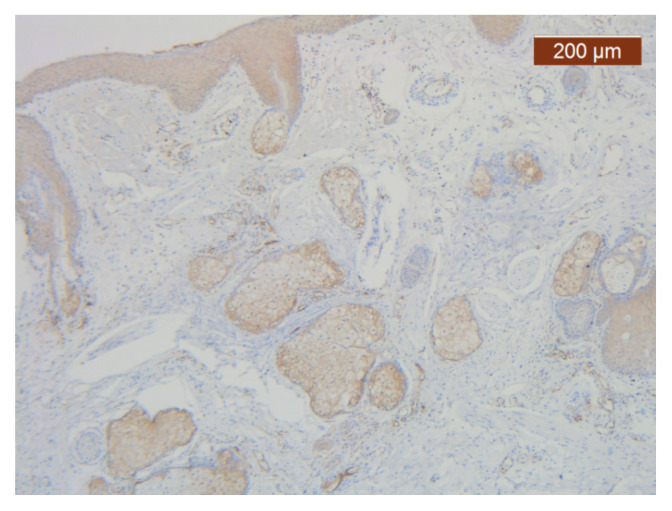
ICAM-2 staining was negative except in blood vessel walls (anti-ICAM-2 ×10 (left), 5 (right)). Staining intensity: weak to moderate.

With the exception of the walls of the large blood vessel captured on the dissected specimen, the expression of the endothelial marker was zero. It is likely that the nutrition of tissues at this level is achieved by microperfusion from the neighboring regions. (Figure 21).

**Figure 22:**
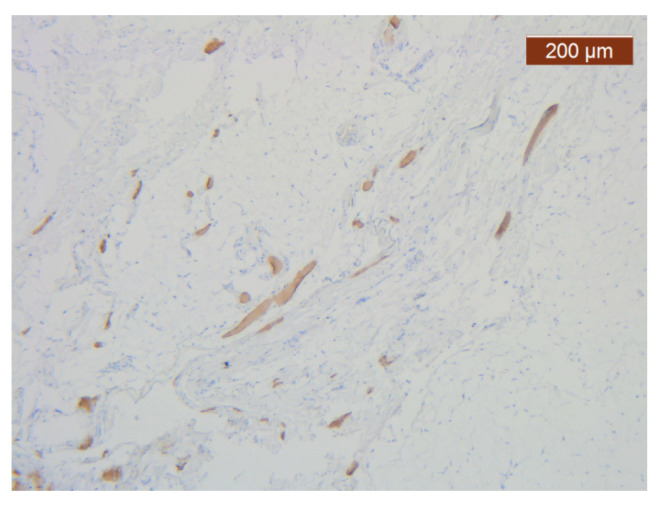
Muscular fibers in deep area of dermis (anti-MyH2 ×5). Staining intensity: moderate.

The muscle fibers are present in the deep layer of the dermis and are not organized in bundles (Figure 22).

In the oral region, the morphohistological and IHC characteristics of the SMAS at the upper and lower labial levels were objectivized. The lateral wall of the oral cavity corresponds to the cheek and has been described previously.

#### 3.3.4. Morphohistological Study and IHC

**Figure 23:**
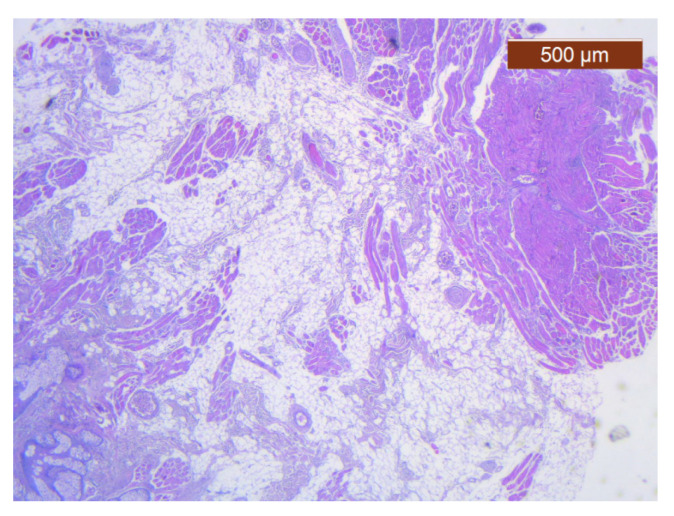
Fibroadipose tissue over the SMAS, well represented with the average thickness of the soft tissue structure (HE ×2.5). Histological piece from biopsy of the lower lip.

**Figure 24:**
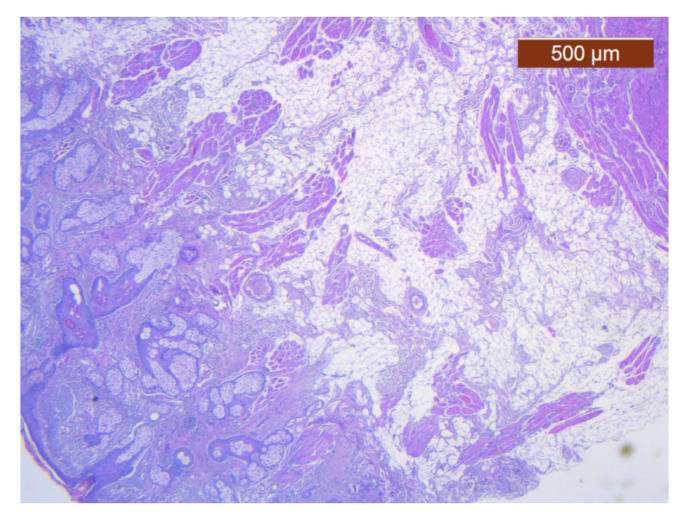
General histological view that showed the depth of the SMAS from the surface of the skin (HE ×2.5). Histological piece from biopsy of the lower lip.

Through the usual staining, a very well-represented layer of collagen can be observed at the levels of both lips (Figure 23 and Figure 24).

**Figure 25:**
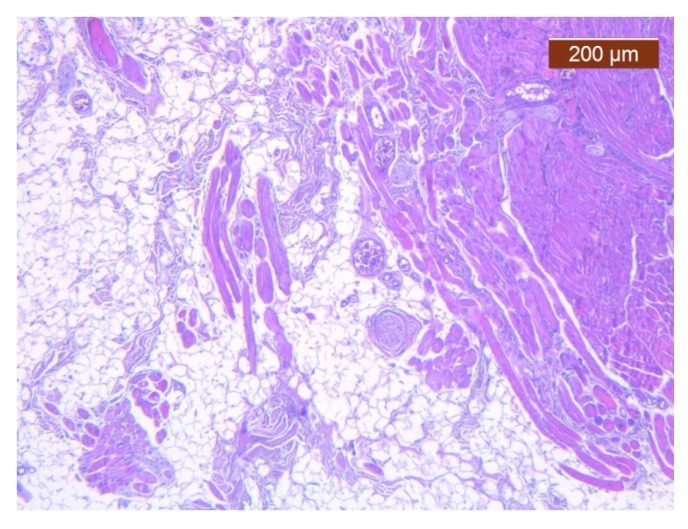
Muscle fibers in the SMAS layer (SMAS fibrous septum). SMAS fibrous septa are arranged perpendicular to the skin and enclose the univacuolar compartments of fat cells (HE ×5). Histological piece from biopsy of the lower lip.

**Figure 26:**
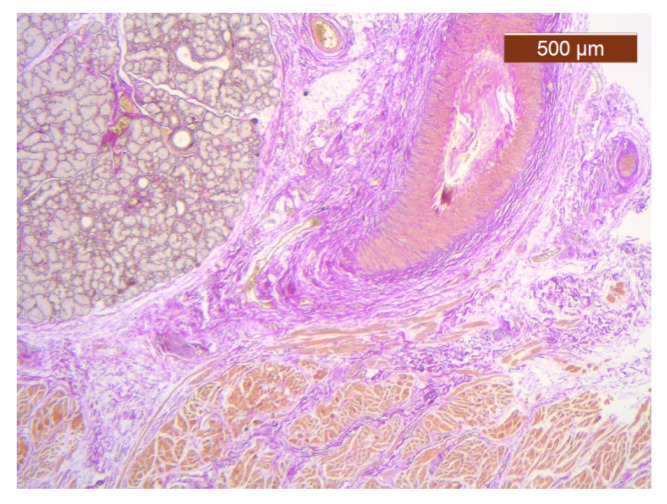
Median layers in the labial region, from top to bottom: salivary glands, skeletal muscle (yellow), and collagen fibers (red) (VG ×2.5). Histological piece from biopsy of the upper lip.

Muscle fibers are also visible and take direct cutaneous insertion, perpendicular to the skin, crossing the superficial fascia (Figure 25 and Figure 26).

**Figure 27:**
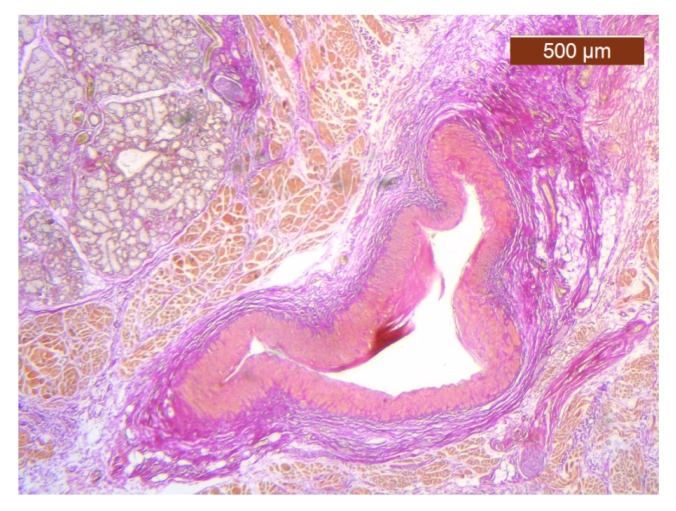
Median layers in the labial region, from top to bottom: salivary glands, skeletal muscle, muscular artery, and perivascular collagen fibers (VG ×2.5). Histological piece from biopsy of the upper lip.

At the labial level, the usual stains could only identify the blood vessels with a caliber of more than 1 mm, which serve the muscle groups and the submucosal glands (Figure 27).

**Figure 28:**
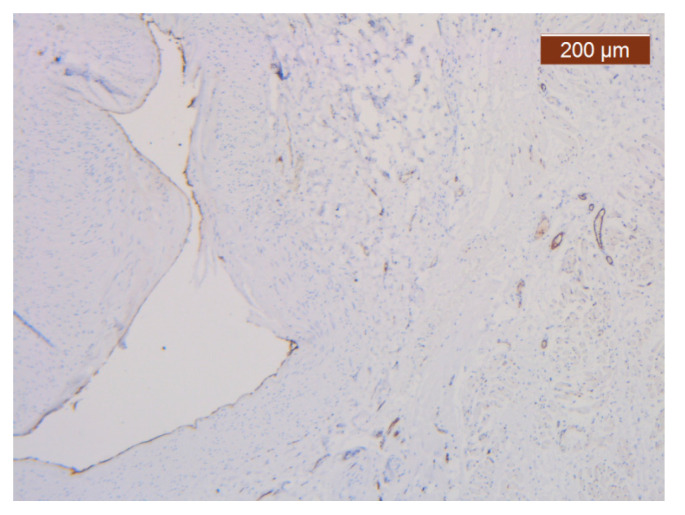
ICAM-2 expression in blood vessel walls and largely absent from the surrounding stroma (anti-ICAM-2 ×5). Color intensity: moderate.

**Figure 29:**
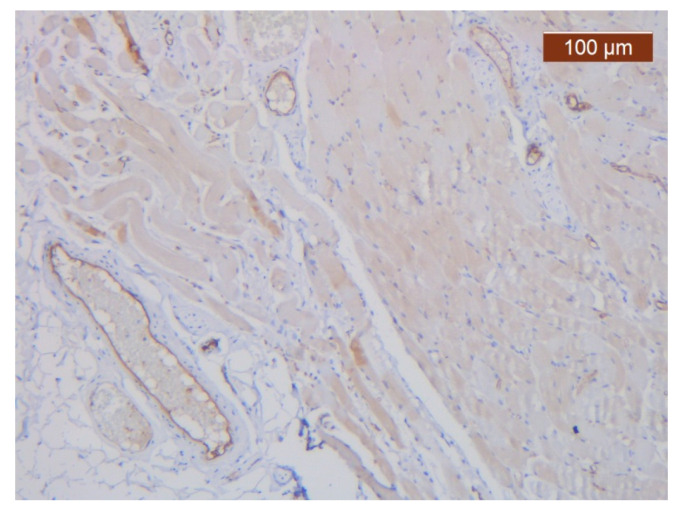
ICAM-2 moderately-intensely expressed in blood vessels of the lower lip (anti-ICAM-2 ×10).

Markers for the vascular endothelium allowed highlighting of a moderately represented SMAS vascular network at the upper (Figure 28) and lower (Figure 29) labial levels.

The intensity of the expression of the ICAM-2 marker was higher in the lower lip (Figure 29) than in the parts collected from the lower lip.

**Figure 30:**
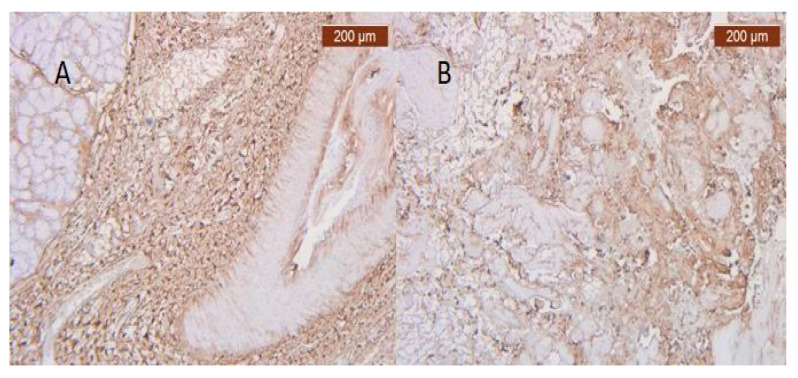
(**A**) Irregular distributions of type III interstitial collagen around the salivary glands (anti-collagen ×5). Color intensity: strong; (**B**) Collagen is abundant in the stroma. The distribution appears predominantly pericellular (anti-collagen III ×5).

Unlike blood vessels, collagen fibers were well represented on specific IHC slides (Figure 30A,B).

**Figure 31:**
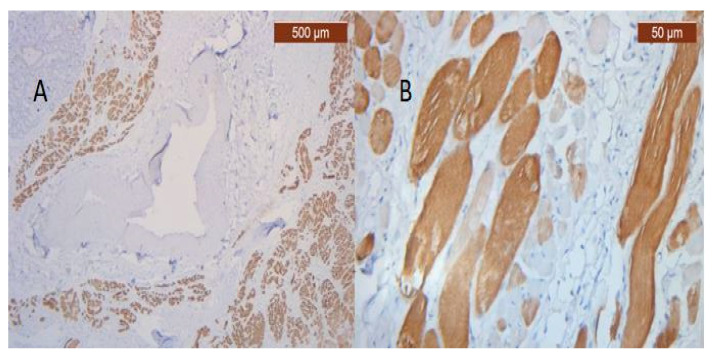
(**A**) Reaction for immunohistochemically positive myosin of muscle fibers in the SMAS fibrous zone (anti-MyH2 ×5). Intensity of coloring at the level of the upper lip: moderate; (**B**) Immunohistochemically positive myosin reaction of muscle fibers in the fibrous area of the SMAS, at the level of the lower lip (anti-MyH2 ×20).

The intensity of muscle-specific IHC marker expression was relatively similar in both lips (Figure 31A,B).

## 4. Results of the Statistical Study

In analyzing the shape of the variable distribution, we focused on the skewness and kurtosis, which allowed us to evaluate the shape of the data distribution.

The coefficient of asymmetry (skewness) expresses the degree of displacement to the left or to the right of the distribution of a variable compared to a normal distribution. The skewness coefficient provides valuable information about the shape of the data distribution and is essential for understanding its behavior in the context of statistical analysis. From the results of our study, this coefficient indicated the degree of differentiation of the elements investigated between the regions of interest.

The coefficient of curvature (kurtosis) indicated which regions exhibited a similar or close level of representation of blood vessels, collagen II fibers, or muscle fibers.

Table 1 shows the statistical value of the Kolmogorov–Smirnov (K–S) test, the number of degrees of freedom (df = 17 and df = 20), and the statistical confidence (Sig. 0.000) for the categorical and numerical independent variables, in relation to the dependent variable “anatomical region“ (nasal, oral). Given that the statistical certainty Sig. 0.000 < 0.05, it can be concluded that the test was statistically significant and that the independent variables did not follow a normal distribution.

The mean rank (i.e., the “Mean Rank” column in Table 2) of the dependent variable, anatomical region, for each group was used to compare the effect of different groups. If the groups in the anatomical region had different scores for each independent variable, then they could be evaluated using the test statistics table showing the results of the Kruskal–Wallis H test, presented in Table 3.

According to the algorithm, the values obtained for each independent variable are centralized in Table 4, where it can be observed that at least one of the z-scores thus obtained, |z_Skewness| > 1 or |z_Kurtosis| > 1, which led to the conclusion that the distribution of the variables differed significantly from a normal distribution.

The distribution for the variable LBV_VHD (Figure 32 and Figure 33) was almost symmetrical, with a small negative asymmetry. Negative kurtosis suggested that the distribution had less pronounced tails than a normal distribution. The Z-values indicated slightly significant deviations from normality. The histogram are for ICAM-2, MyoH2, LTD, LVD, and LBV_VHD (Figure 34, Figure 35, Figure 36, Figure 37, Figure 38, Figure 39, Figure 40, Figure 41, Figure 42 and Figure 43).

**Figure 32:**
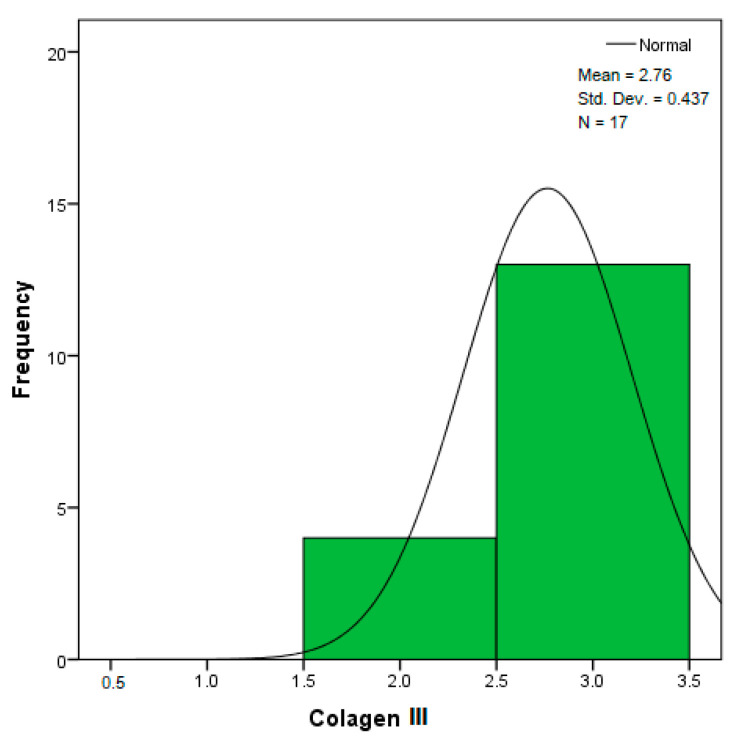
Histogram for the variable collagen III according to the dependent variable nasal anatomical region.

**Figure 33:**
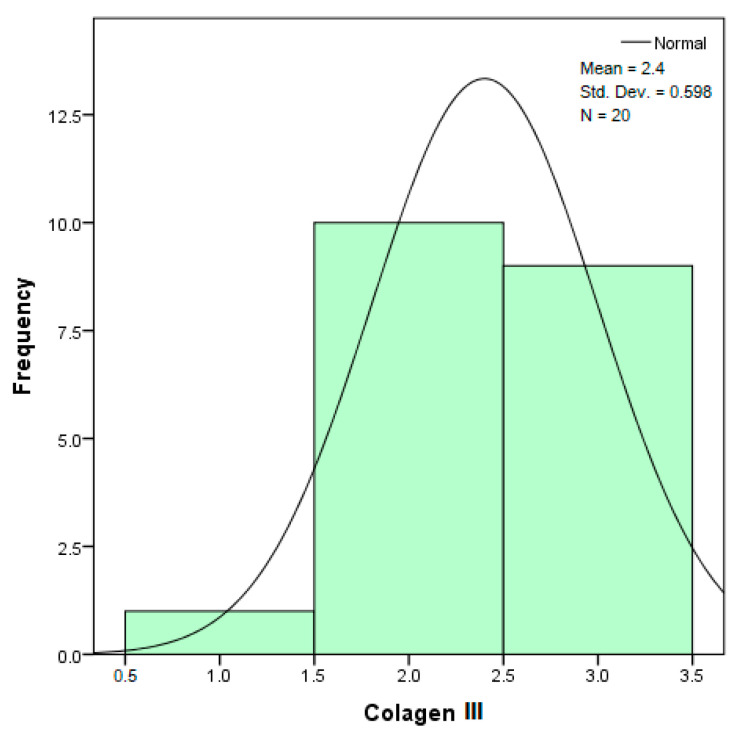
Histogram for the variable collagen III according to the dependent variable oral anatomical region.

From Figure 32 and Figure 33, it can be seen that the distribution for the variable collagen III was asymmetric to the left (negative skewness), indicating that most values were high, but there were some small values that pulled the average down. Negative kurtosis suggested a distribution with less pronounced tails than a normal distribution. The Z-values for skewness and kurtosis were below the 1.96 threshold, suggesting that the deviations from normality were not very significant.

**Figure 34:**
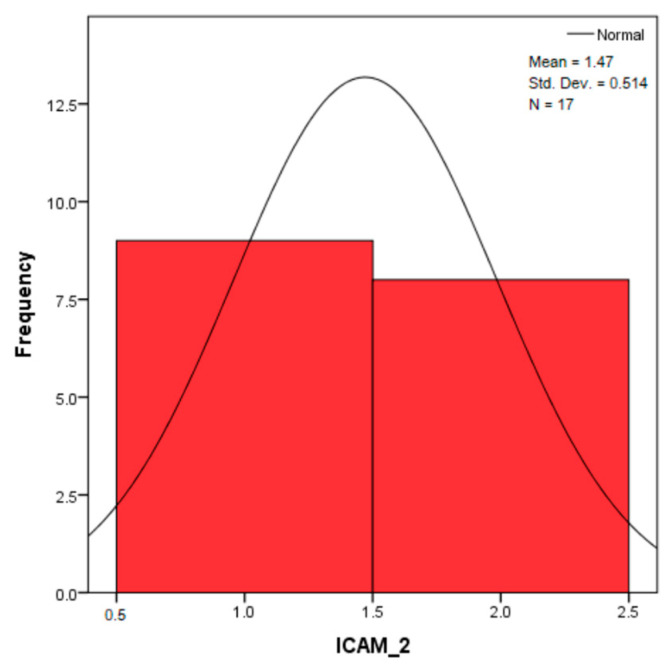
Histogram for the variable ICAM-2 according to the dependent variable nasal anatomical region.

**Figure 35:**
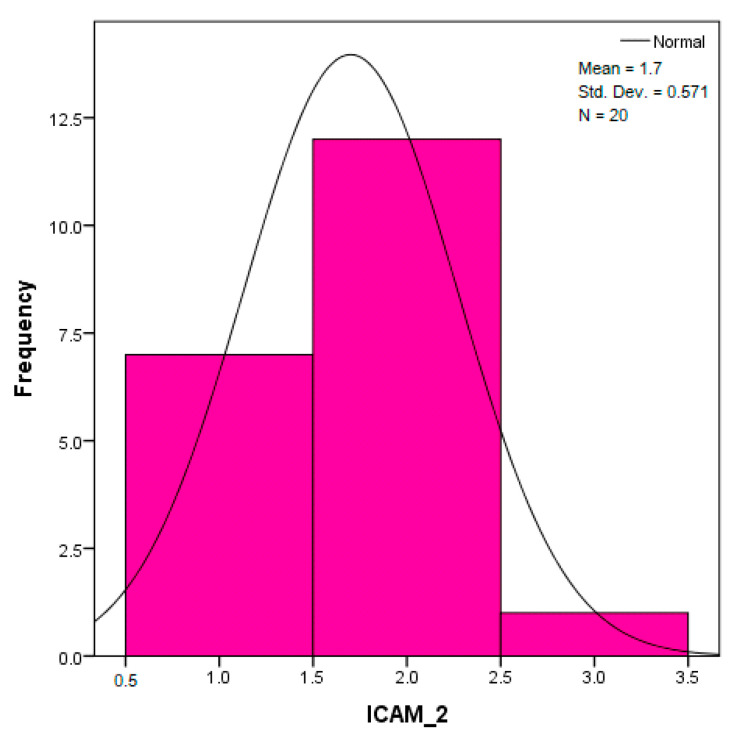
Histogram for the variable ICAM-2 according to the dependent variable oral anatomical region.

The distribution for ICAM-2 (Figure 34 and Figure 35) was almost symmetrical (skewness close to 0), indicating a relatively normal distribution. Negative kurtosis suggested less extended tails than those of a normal distribution. The Z-values for skewness and kurtosis suggested that these deviations were slightly above the significance limits.

**Figure 36:**
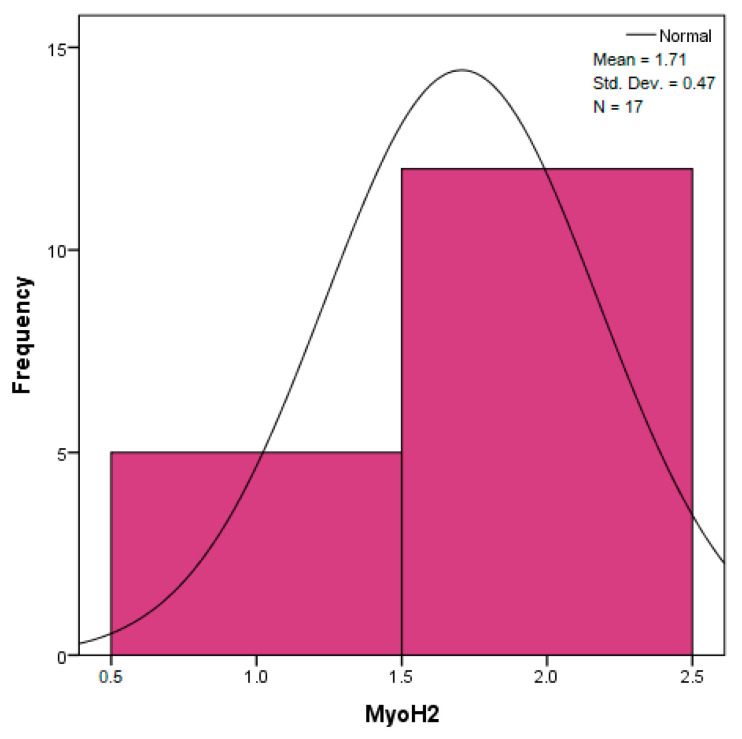
Histogram for the variable MyoH2 according to the dependent variable nasal anatomical region.

**Figure 37:**
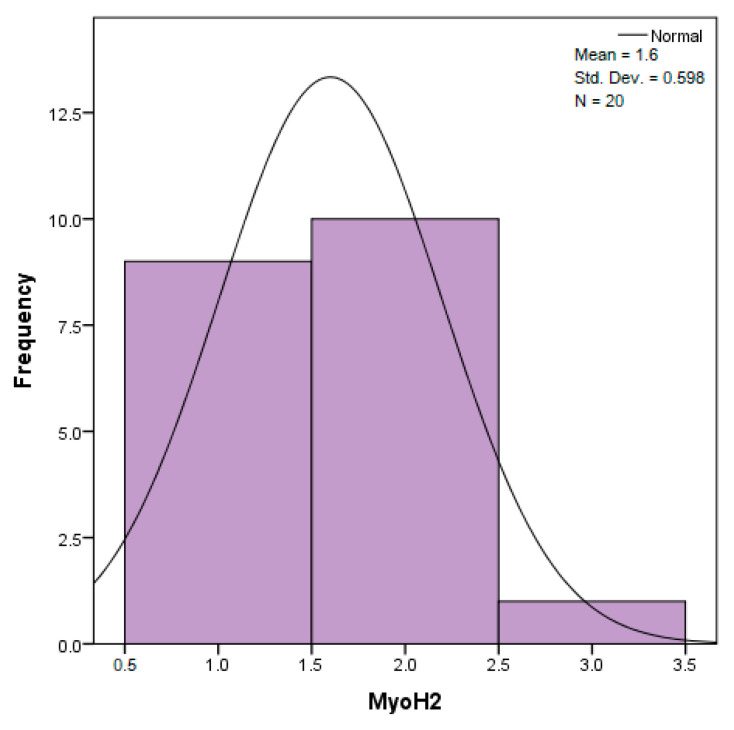
Histogram for the variable MyoH2 according to the dependent variable oral anatomical region.

The distribution for MyoH2 (Figure 36 and Figure 37) was almost symmetrical, with very little asymmetry. Negative kurtosis indicated a distribution with tails less extended than a normal distribution. The Z-values were below the threshold of 1.96, which suggested that the distribution was fairly close to normal.

**Figure 38:**
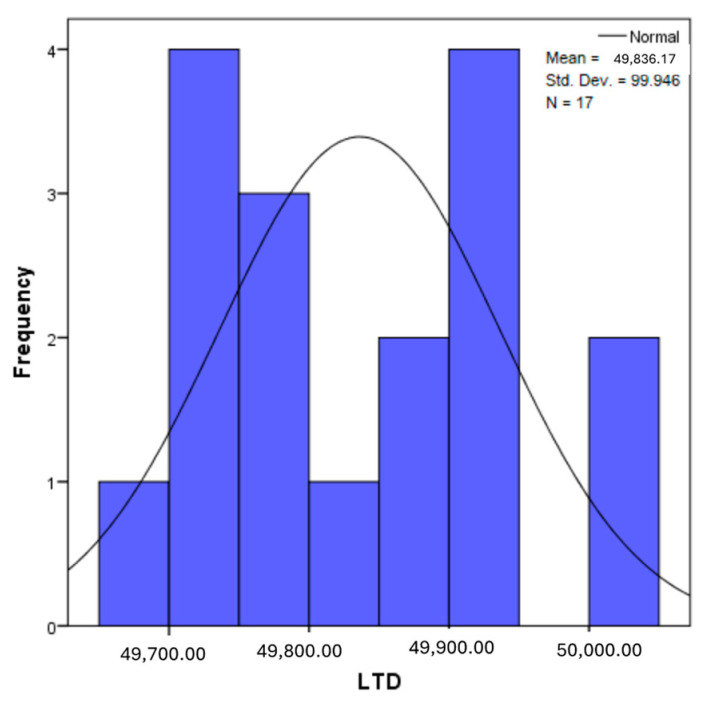
Histogram for the variable LTD according to the dependent variable nasal anatomical region.

**Figure 39:**
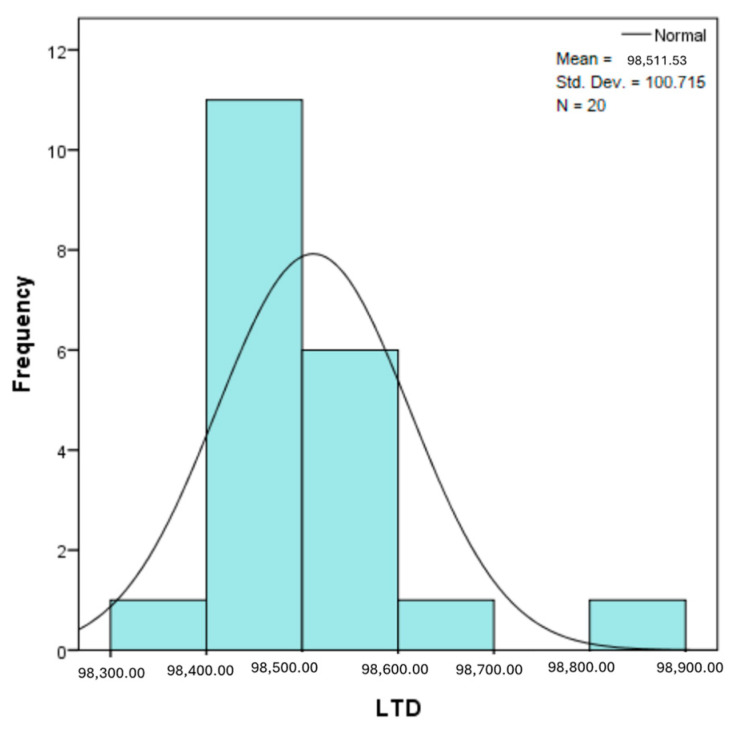
Histogram for the variable LTD according to the dependent variable oral anatomical region.

From Figure 38 and Figure 39, it can be seen that the distribution for the variable LTD showed a slight positive asymmetry, which indicated a predominance of small values, but without large deviations. Significant negative kurtosis suggested less extended tails than in a normal distribution. The Z-values for skewness and kurtosis were below the 1.96 threshold, indicating moderate deviations from normality.

**Figure 40:**
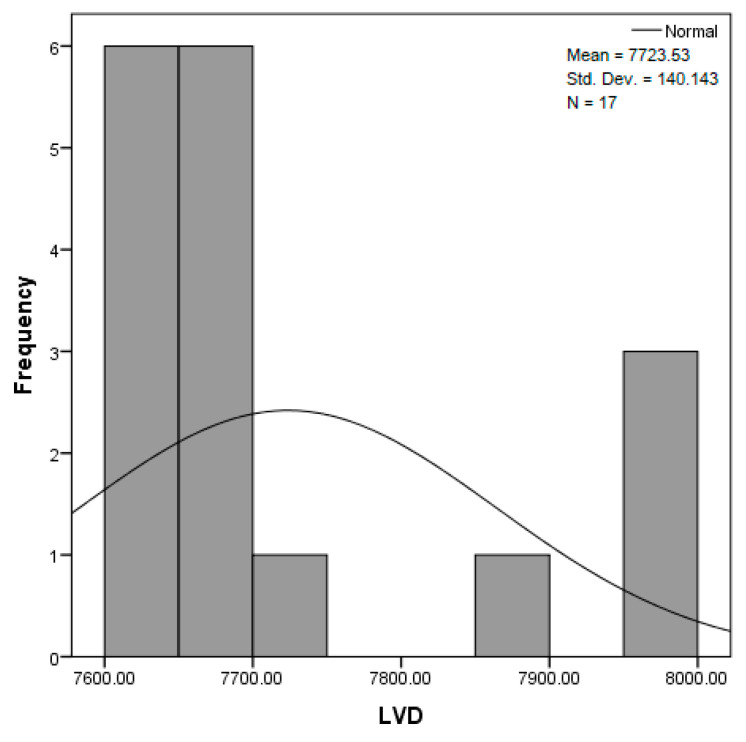
Histogram for the variable LVD according to the dependent variable nasal anatomical region.

**Figure 41:**
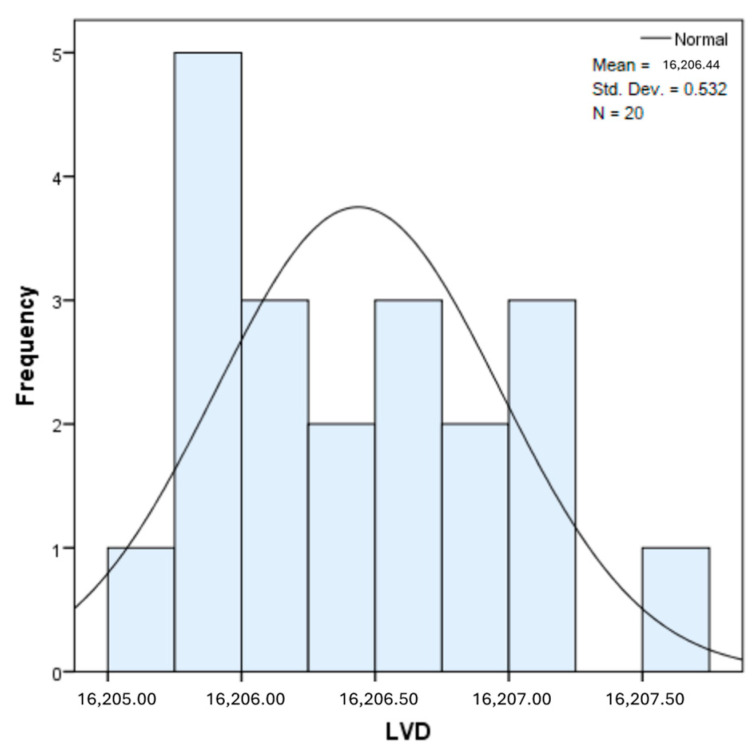
Histogram for the variable LVD according to the dependent variable oral anatomical region.

The distribution for the variable LVD (Figure 40 and Figure 41) showed a slight positive asymmetry, suggesting a predominance of small values, but without major deviations. Negative kurtosis indicated less extended tails than those of a normal distribution. The Z-values suggested that the deviations from normality were not very significant.

## 5. Discussion

This research successfully achieved its primary objective of directly visualizing the “intrinsic vascular apparatus of the SMAS”. The dermis represents an extremely heterogeneous tissue compartment in human skin, given its fibroblast content and extracellular matrix structure. Its segmentation into two biologically distinct territories (i.e., superficial papillary dermis and deeper reticular dermis) occurs during embryonic development at 12 weeks of gestation in humans [32]. These morphological differences lead to significant functional variations, even in the same region. The main structural features of these dermal types and topographies are related to collagen cross-linking and elastin network organization. They are dynamic features that undergo constant evolution during intrauterine and postnatal life [33]. These phenomena involve major differences regarding the vascularization of these regions.

Our study results allowed us to identify the distinctive regional characteristics of the SMAS, particularly of the blood vessels that cross it. An important aspect is related to the characteristics of the route, appearance, and morphometry of these vessels, which implicitly vary depending on the main distribution territory. Thus, the blood vessels with a majority distribution toward the skin have a well-differentiated and delimited trans-SMAS path. The distribution vessels of the SMAS are derived from the cutaneous ones, as retrograde branches or directly approaching the SMAS layers coming from the deep layer and passing through the muscle fascicles.

Intrauterine life is considered to explain the different behaviors observed among the connective, nervous, and vascular tissues in each nasal subregion examined. In our study, we managed to correlate the micro-CT method with IHC techniques, which have not been used until now in the study of SMAS microvascularization. Our morphometric and topographical results can be correlated with future functional studies to provide a complete picture.

In the nasal SMAS architecture, we found aspects that correspond mostly to the type 2 SMAS, as described by Ghassemi (2003) [8]. In the classical anatomy of the SMAS, it is considered that this structure is crossed by blood vessels that branch inside it and go to the deep layer of the skin. Concurrently, it is considered that the SMAS bed is relatively poorly or even critically vascularized [34,35,36].

Micro-CT, initially developed for assessing bone microarchitecture, is now widely used for imaging soft tissues, particularly blood vessels. Its main advantage is the ability to visualize capillary-sized vessels, though the technique remains somewhat subjective, semi-quantitative, and not specifically designed for vascular imaging [37,38,39].

We managed to individualize a study protocol on the microperfusion vascularization of the SMAS based on the experience accumulated in the abovementioned studies. Moreover, the research demonstrates a morphological image of the vascularization and microperfusion of the SMAS and the dermis. The micro-CT study results consolidate and detail those obtained in the histological studies. Our study shows that the three nose subregions studied have different morphological and, implicitly, functional characteristics of the SMAS and its blood supply.

Intercellular adhesion molecule (ICAM)-2 is located at the level of endothelial junctions and is involved in leukocyte recruitment and neoangiogenesis processes [40,41]. Endothelial integrity is maintained by endothelial junctions, which also regulate vascular homeostasis. The endothelial junctions mediate cell trafficking in and out of the tissues, cell–cell contact, and endothelial survival and apoptosis. These junctions have vascular endothelial-cadherin and the CD31/platelet endothelial cell adhesion molecule, which mediate contact between adjacent endothelial cells. They also control leukocyte transmigration and angiogenesis.

The leukocyte adhesion molecule intercellular adhesion molecule 2 (ICAM-2) is also expressed in these junctions. Recent data have shown that ICAM-2 regulates angiogenesis via several mechanisms, including survival, cell migration, and small guanosine triphosphatase (GTPase) Rac activation [42]. These recent findings allowed us to use ICAM-2 to detect the endothelial cells in our research.

Regarding the materials used in the morphohistological study, this method allowed us to use non-formalized specimens. Formalized and fresh–frozen cadavers are, in general, more easily accessible to researchers than fresh, unfrozen cadavers; however, the embalming process, through the substances used and the time of exposure to them, changes collagen, muscle fiber, and blood vessel structures [43,44]. The technique used to collect specimens was dictated by the main objective of the surgical intervention received by the patient.

The choice of the I-CAM2 marker is justified by the fact that the expression of intercellular adhesion molecule 2 (ICAM-2) in adult tissues is present on all vascular endothelial cells. Recent studies have shown that the in vivo endothelium-specific activity of the human ICAM-2 promoter is contained in a small (0.33 kilobase (kbp) 5′-region of the gene. In vitro and in vivo, in these regions, the ICAM-2 promoter is TATA-less and the transcription in endothelial cells is initiated at four sites [45]. This suggests that transcription from the ICAM-2 promoter in endothelial cells is regulated by the interaction of several factors and provides a solid basis for a detailed analysis of the presence of endothelial tissue in the studied sample [45]. In our study, we found endothelial cells in all of our samples. The organization of these vascular structures, as well as their relationship with collagen II fibers and fibrin, vary significantly from one region to another. The results indicate the presence of blood vessels proper to the SMAS layer in all studied regions, but with significant differences related to their size, density, and orientation.

Considering these, we discovered that each topographic layer of the SMAS presents distinct vascular characteristics. Thus, the stroma is moderately vascularized by its own blood vessels only in the subregion corresponding to the wing of the nose. At the level of the nasal pyramid and nasolabial fold, this is performed, most likely by microperfusion.

The presence of muscle fibers also has different aspects. At the level of the nasal pyramid, they are predominantly found between two dense layers of collagen, whereas at the level of the wing of the nose, they cross the entire supporting conjunctival network. At the level of the nasolabial fold, the muscle fibers are dispersed in the deep dermis [11]. Our results highlight similar aspects.

We have also found that, in all the three subregions, the collagen fibers are densely represented at the stroma level. In the nasal pyramid, they form strong connective septa at this level. In the medial part of the nasolabial groove, collagen III fibers form a dense network throughout the SMAS thickness.

In all of these subregions, the common characteristics of SMAS vascularization distribution can be observed. The blood vessels belonging to the SMAS are found, especially in the topographic layer of the mimic muscle fibers. They have connective sheaths. The weak or moderate reaction to the IHC marker I-CAM2 showed weak activity regarding the processes of neoangiogenesis and transmembrane transport, especially at the levels of the nasal pyramid and nasolabial fold. These represent aspects we have not found anywhere in the literature.

Concurrently, the presence of medium blood vessel IHC response, represented only in the intermediate layer of the SMAS at this level, underlines the poor superficial vascularization. Even so, these findings of our study show that a complex fibrous structure, which is the nasolabial fold, has a thinner but organized vascular network.

Vascular restoration must be combined with aesthetic restoration but first with functional recovery of the SMAS [46]. This raises special problems regarding vascular and implicit functional restoration of the constituents of this complex musculoaponeurotic system. In achieving this goal, it is mandatory to consider the topographical and morphometric aspects of SMAS vascularization and its source of origin.

The morphohistological characteristics of the oral region must be considered in the functional context of the muscular apparatus attached to the orbicularis oris muscle, in the concept of the “buccinator apparatus” and its participation in regional biomechanics. Medial and inferior to the nasolabial sulcus, the zygomatic muscles, the levator of the upper lip, and the levator of the angle of the mouth take dermal insertion. In addition, the orbicularis oculi muscle adheres intimately, passing through a fine network of type III collagen, to the deep face of the dermis.

A well-represented subcutaneous adipose layer, including the zygomatic fat pad, is evident on the lateral side of this groove. This adipose layer provides the superficial soft tissues at this level with an antigravity support. With advancing age and quantitative loss of this layer, ptosis occurs and the groove deepens.

The zygomatic ligament also affects this region. It is located in the sub-SMAS topographic plane, and it antigravitationally supports the superficial soft tissues by anchoring the SMAS to the periosteum of the region and sends extensions on the fascia of the zygomatic muscles to their oral insertion. The zygomatic ligament, together with the homonymous muscles and their fascia, are part of the McGregor complex, inserting on the deep part of the overlying dermis. Consequently, this musculo-ligamentous complex plays a key role in the movements made at the level of the mouth [47,48].

The anatomical study, supported by the surgical one, confirms that the nasolabial fold represents the transition limit between the skin territories of the cheek and the upper lip being one of the key points of facial surgery. IHC staining for the vascular endothelium, together with the µCT study, show a fine vascular network proper to the SMAS, including at this level.

At the level of the lower lip, the existence of a histologically distinct SMAS layer is demonstrated. At this level, type III collagen fibers fuse with muscle fibers and contain a well-represented network of blood vessels.

The main feature of the oral region is the existence of a sub-SMAS space, in which adipose tissue is located. This space can represent a way for an infection to spread to neighboring regions. Collagen fibers form connective tracts that separate the fat lobules at this level and can limit the spread of an infection.

Another important aspect is that this study finds a unique SMAS layer at the oral level and in the surrounding regions, contrary to the controversies so far [49]. The upper lip exhibits a cytoarchitectural and microvascular SMAS organization similar to that of the lower lip.

All of these results confirm that there is a continuation of the SMAS with the superficial layer of the orbicularis oculi muscle, suggesting that this layer represents the SMAS of the upper lip, separate from the overlying fascial layer.

Anatomical, surgical, and laboratory studies suggest that the SMAS tunnels the facial nerve bundles, even helping to map them. Vascularization and innervation of the skin of the face is achieved directly, trans-SMAS, and maintaining its integrity guarantees the success of a skin graft [50]. Its existence and knowledge allows for surgical sectioning of the fixation formations to inactivate a free tension, which allows the mobilization and repositioning of a skin fold for reconstruction or facelift. The presence of the vascular network in the center of the SMAS (discovered by us) indicates the need to perform deeper incisions to preserve the revascularization conditions of the skin at this level. The topography of the muscle fibers in the vicinity of the vascular network reveals the possibility of an easy spontaneous hemostasis, including in this region.

All of the observations and measurements made support finding the optimal surgical procedures for excision of a tumor, orofacial reconstruction, or performing different types of injections.

The research carried out supports the existence of microvascularization of the SMAS in the oral region, as a unitary structure, specific to this region.

The variable collagen III shows an asymmetric distribution to the left, having a flatter shape than a normal one. In general, the variable ICAM-2 shows a relatively balanced and uniform distribution, with minimal skewness and a more flattened shape compared to the normal distribution. The variable MyoH2 shows a relatively balanced and almost symmetrical distribution.

By applying the Shapiro–Wilk (S–W) test, the statistical certainty Sig. 0.000 < 0.05, and normality tests indicate that non-parametric methods (such as the Kruskal–Wallis test) should be used to check whether the samples come from the same distribution. By applying the Kruskal–Wallis test, it is observed that the estimated value of the chi-square statistic for each independent variable is significant at a confidence level of 95% because of the value of Asymp. Sig. < 0.05, with the exception of the variables ICAM-2 and MyoH2.

The Kruskal–Wallis test indicates the existence of significant differences between the nasal and oral regions for all analyzed variables, such as:

- The values of the variable collagen and MyoH2 are higher in the nasal region than in the oral region;
- The values of the variable LTD, LVD, and LBV_VHD are significantly higher in the oral region than in the nasal one;
- ICAM_2 has higher values in the oral region than in the nasal region.

**Figure 42:**
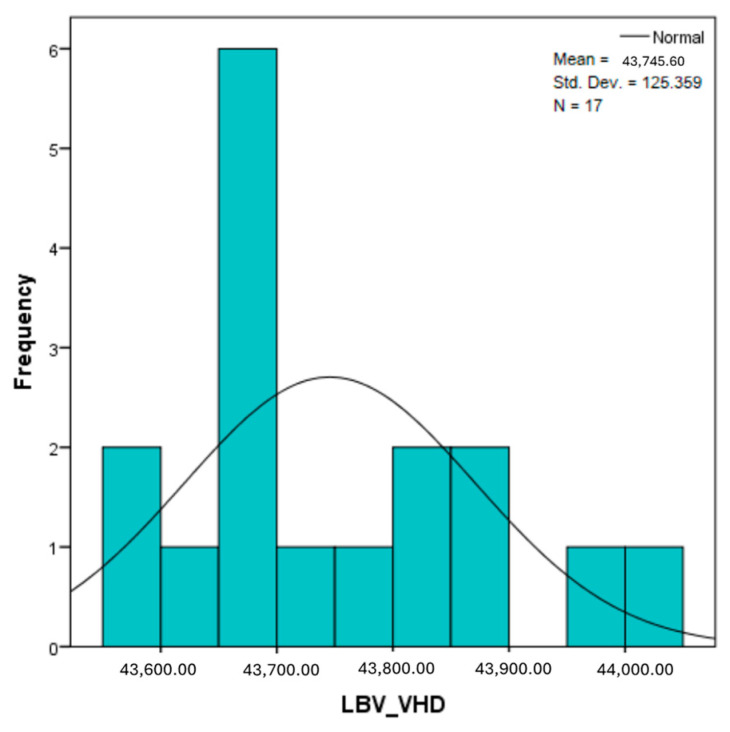
Histogram for the variable LBV_VHD according to the dependent variable nasal anatomical region.

**Figure 43:**
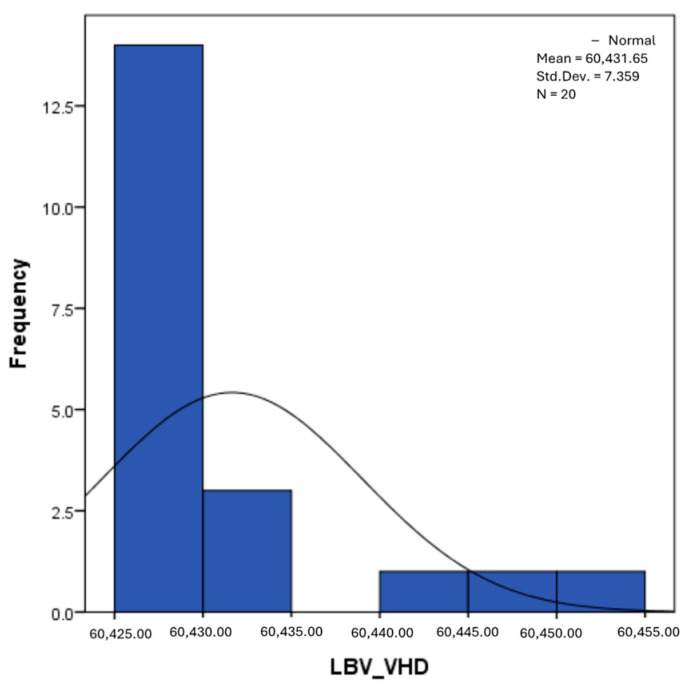
Histogram for the variable LBV_VHD according to the dependent variable oral anatomical region.

The verification of the normality of the distribution of the results obtained for the variables included in the study was also carried out by graphical methods. The most common graphical technique is the representation of data in the form of a histogram, illustrated in Figure 33, Figure 34, Figure 35, Figure 36, Figure 37, Figure 38, Figure 39, Figure 40, Figure 41, Figure 42 and Figure 43. From these figures, it can be seen that the data frequency distribution for each variable does not follow a normal distribution law, being characterized by asymmetry either to the left or to the right.

In an asymmetric distribution to the left (negative skewness distribution, the skewness statistic has the value (−0.797), for the variable collagen III, according to Table 2), high scores predominate (3+ large amounts/strong intensity). In this case, the modulus is the rightmost value in the data string, and the median is greater than the mean. The histogram shows an accumulation of frequencies on the right side of the graph, with a spread of values to the left. As seen in the study, the distribution of the variable collagen III is skewed to the left, indicating that most values for the amount or intensity of collagen III are high, but there are a few isolated cases with very low values. This suggests that, in general, collagen III is present in large amounts in the samples, although some samples are significantly deficient.

In an asymmetric distribution to the right (positive skewness distribution, the skewness statistic has the value (0.076), for the variable MyoH2, according to Table 2), low scores predominate (2+ moderate amounts/moderate intensity). In this case, the modulus is the leftmost value in the data string, and the median is greater than the mean. From the analysis of the precision of the indicators of central tendency, we know that in a series of data where we encounter extremely high scores, the average tends to value them. This is graphically illustrated in Figure 10, and the relationship existing in such a distribution is: Mo < Me < m. This is the characteristic relationship of a positive skewed distribution. Because the statistical safety Sig. 0.000 < 0.05 was obtained by both statistical tests, according to Table 1, the null hypothesis was rejected (i.e., it is unlikely to obtain such data assuming that they are normally distributed).

This research comes with a number of particularly important novelties, which are summarized in order of their scientific importance:

- The discovery of an intra-SMAS vascular plexus, in all midfacial regions;
- Highlighting the SMAS and its continuity in all studied regions;
- The simultaneous use of three different immunohistochemical markers, for vascular endothelium, muscle fibers, and type III collagen;
- Integrating the results of observational studies in a morphometric manner and their statistical interpretation;
- Interpreting the clinical impact of SMAS microvascular morphometry.

## 6. Conclusions

The results confirm the existence of a vascular network (plexus) within the SMAS layer, characterized by the spatial distribution and topographic arrangement of SMAS blood vessels, and provide a quantitative assessment of their caliber and density. Collagen distribution varies significantly by region. In the nasal pyramid is trilamellar collagen. In the alar areas, collagen is present but with notable irregularities, especially in terms of collagen III, reflecting a dense stromal distribution. The nasolabial fold region shows the densest and most organized collagen meshwork, while the oral region, particularly the lips, also has a robust collagen layer arranged above the SMAS. Staining intensities and collagen structures differ, reflecting the diverse functional and structural demands of each region. Furthermore, we identified and characterized blood vessels in anatomical structures previously deemed avascular, evaluated the functional and clinical implications of the findings, and laid the groundwork for future research in this area. In the future, detailed related studies on laboratory animals are required. Moreover, it is necessary to evaluate the presence of the nerve threads in these regions and their relationship with muscular and vascular threads.

## Figures and Tables

**Table 1:** Normality tests for the independent variables.

Variable	Anat_reg	Kolmogorov–Smirnov			Shapiro–Wilk		
		Statistic	df	Sig.	Statistic	df	Sig.
LTD	nazal	0.163	17	0.200	0.929	17	0.207
	oral	0.314	20	0.000	0.627	20	0.000
Colagen	nazal	0.469	17	0.000	0.533	17	0.000
	oral	0.298	20	0.000	0.744	20	0.000
ICAM_2	nazal	0.349	17	0.000	0.642	17	0.000
	oral	0.350	20	0.000	0.736	20	0.000
MyoH2	nazal	0.440	17	0.000	0.579	17	0.000
	oral	0.298	20	0.000	0.744	20	0.000
LVD	nazal	0.320	17	0.000	0.732	17	0.000
	oral	0.112	20	0.200	0.953	20	0.419
LBV_VHD	nazal	0.230	17	0.018	0.910	17	0.098
	oral	0.390	20	0.000	0.584	20	0.000

**Table 2:** Ranks.

Variable	Anat_reg	N	Mean Rank
Colagen	nazal	17	22.26
	oral	20	16.23
	Total	37	
ICAM_2	nazal	17	16.76
	oral	20	20.90
	Total	37	
MyoH2	nazal	17	20.21
	oral	20	17.98
	Total	37	
LTD	nazal	17	9.00
	oral	20	27.50
	Total	37	
LVD	nazal	17	9.00
	oral	20	27.50
	Total	37	
LBV_VHD	nazal	17	9.00
	oral	20	27.50
	Total	37	

**Table 3:** Test Statistics.

	Colagen	ICAM_2	MyoH2	LTD	LVD	LBV_VHD
Chi-Square	3.886	1.728	0.530	26.842	26.845	26.842
df	1	1	1	1	1	1
Asymp. Sig.	0.039	0.189	0.467	0.000	0.000	0.000

**Table 4:** Descriptive statistics for questionnaire items.

Variable	Mean	Std. Deviation	Skewness (Eroarea Standard)	Kurtosis (Eroarea Standard)	$\left|Z_{Skewness}\right|$	$\left|Z_{Kurtosis}\right|$
Colagen III	2.57	0.555	−0.797 (0.388)	−0.406 (0.759)	0.438	1.658
ICAM-2	1.59	0.551	−0.131 (0.388)	−0.969 (0.759)	0.338	1.129
MyoH2	1.65	0.538	−0.076 (0.388)	−0.864 (0.759)	0.196	1.067
LTD	76,147.17	24,592.35	0.170 (0.388)	−2.087 (0.759)	0.438	1.658
LVD	12,308.88	4286.82	0.171 (0.388)	−2.085 (0.759)	0.441	1.657
LBV_VHD	52,765.09	8430.67	−0.170 (0.388)	−2.087 (0.759)	0.438	1.658

## Data Availability

Data are contained within the article.
